# Inhibition of iron‐induced cofilin activation and inflammation in microglia by a novel cofilin inhibitor

**DOI:** 10.1111/jnc.16260

**Published:** 2024-11-18

**Authors:** Faheem Shehjar, Antonisamy William James, Reetika Mahajan, Zahoor A. Shah

**Affiliations:** ^1^ Department of Medicinal and Biological Chemistry College of Pharmacy and Pharmaceutical Sciences Toledo Ohio USA

**Keywords:** cofilin activation, iron overload, microglial activation, neuroinflammation, NF‐κB

## Abstract

Neuroinflammatory conditions linked to iron dysregulation pose significant challenges in neurodegenerative diseases. Iron‐loaded microglia are observed in the brains of patients with various neuroinflammatory conditions, yet how iron overload affects microglial function and contributes to various neuroinflammatory processes is poorly understood. This in vitro study elucidates the relationship between excess iron, cofilin activation, and microglial function, shedding light on potential therapeutic avenues. Iron overload was induced in Human Microglial Clone 3 cells using ferrous sulfate, and the expressions of ferritin heavy chain, ferritin light chain, divalent metal transporter 1, cofilin, p‐cofilin, nuclear factor‐κB (NF‐κB), and various inflammatory cytokines were analyzed using real‐time quantitative polymerase chain reaction, immunocytochemistry, Western blotting, and enzyme‐linked immunosorbent assay. Results revealed a notable increase in cofilin, NF‐κB, and inflammatory cytokine expression levels following excess iron exposure. Moreover, treatment with deferoxamine (DFX), a known iron chelator, and a novel cofilin inhibitor (CI) synthesized in our laboratory demonstrate a mitigating effect on iron‐induced cofilin expression. Furthermore, both DFX and CI exhibit promising outcomes in mitigating the inflammatory consequences of excess iron, including the expression of pro‐inflammatory cytokines and NF‐κB activation. These findings suggest that both DFX and CI can potentially alleviate microglia‐induced neuroinflammation by targeting both iron dysregulation and cofilin‐mediated pathways. Overall, this study provides valuable insights into iron‐induced cofilin activation and microglial activation, offering avenues for potential targeted therapies for neuroinflammatory conditions associated with iron and cofilin dysregulation in neurodegenerative diseases.

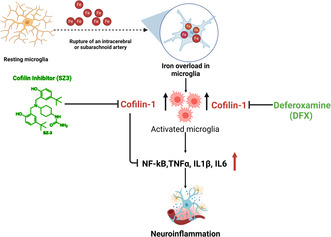

## Graphical abstract

Iron dysregulation is a common feature in neurodegenerative diseases, especially in the microglia. This study investigates the impact of iron overload on microglial function, focusing on cofilin activation, an essential protein in cytoskeletal dynamics, and its role in neuroinflammation. The novel findings demonstrate that excess iron (FeSO_4_) increases cofilin expression, associated with enhanced inflammatory cytokine production and activation in the nuclear factor‐κB (NF‐κB) pathway. Treatments with deferoxamine (an iron chelator) and a novel cofilin inhibitor (CI) successfully mitigate these effects, offering potential therapeutic avenues for neuroinflammatory diseases. These results emphasize the dual targeting of iron dysregulation and cofilin pathways as a promising approach for managing neurodegeneration linked to iron overload.

**Abbreviations**
ATCC: American Type Culture Collection
CCK‐8: Cell Counting Kit‐8
CI: cofilin inhibitor
DFX: deferoxamine
DMT1: Divalent Metal Transporter 1
ELISA: enzyme‐linked immunosorbent assay
EMEM: Eagle's Minimum Essential Medium
FBS: fetal bovine serum
FeSO_4_
: ferrous sulfate
FTH: ferritin heavy chain
FTL: ferritin light chain
HMC‐3: human microglial clone 3
IL‐12: interleukin 12
IL‐1β: interleukin 1 beta
IL‐6: interleukin 6
NF‐κB: nuclear factor kappa B
PBS: phosphate‐buffered saline
p‐cofilin: phosphorylated cofilin
PVDF: polyvinylidene fluoride
RT‐qPCR: real‐time quantitative polymerase chain reaction
SDS‐PAGE: sodium dodecyl sulfate‐polyacrylamide gel electrophoresis
SEM: standard error of the mean
TBST: Tris‐buffered saline with Tween 20
TNF‐α: tumor necrosis factor‐alpha

## 1 INTRODUCTION

Microglia, the resident immune cells of the central nervous system (CNS), have garnered increasing attention for their pivotal roles in maintaining brain homeostasis, synaptic plasticity, and neuroinflammation (Borst et al., 2021). Once considered passive bystanders, these versatile cells are now recognized as dynamic players in physiological and pathological conditions (Prinz et al., 2019). Ability of microglia to rapidly respond to CNS insults, clear cellular debris, modulate synaptic connections, and regulate neuroinflammation underscores their significance in brain health and disease (Cowan et al., 2022). Neuroinflammation is a critical factor in the pathogenesis of various neurological disorders, and understanding the molecular mechanisms underlying inflammatory responses in microglia is essential for developing targeted therapeutic interventions (Shao et al., 2022).

Microglia are sensitive to changes in their microenvironment, including alterations in iron homeostasis (Gao et al., 2023; Kenkhuis et al., 2021). While iron is an essential element for cellular functions, its dysregulation can lead to the generation of reactive oxygen species and the activation of pro‐inflammatory pathways (Chen et al., 2023). Excessive iron has been implicated in neuroinflammation, oxidative stress, and neuronal damage in various brain‐related pathologies such as Alzheimer's disease (AD), Parkinson's disease (PD), and stroke, among others (Levi et al., 2024).

Cofilin, a protein crucial for regulating actin dynamics, has recently emerged as a key mediator in the interplay of microglia and neuroinflammation (Alhadidi et al., 2016; Alsegiani, 2022; Shehjar et al., 2024). Studies suggest that cofilin activation is intricately linked to inflammatory responses in microglia, leading to neurodegeneration (Kenkhuis et al., 2021; Yan et al., 2022).

Keeping in view the above facts, it is clearly established that both iron and cofilin are involved in microglial activation and homeostasis (Alhadidi & Shah, 2018; Liu et al., 2022; Long et al., 2022; Ryan et al., 2023). However, the specific effects of excess iron on cofilin dynamics and the subsequent inflammatory consequences in microglia remain to be fully elucidated. To address this knowledge gap, our study employed an in vitro model using human microglia cell line‐3 (HMC‐3). These cells were treated with ferrous sulfate (FeSO_4_), a common form of iron, to simulate conditions of iron overload. We aimed to investigate the impact of excess iron on cofilin expression, microglial activation, and downstream inflammatory responses. Additionally, we explored potential therapeutic strategies by utilizing a common iron chelator deferoxamine (DFX) and a novel first‐in‐class cofilin inhibitor (CI) synthesized in our lab. DFX, known for its iron‐sequestering properties, was employed to assess its efficacy in mitigating the effects of excess iron on microglia. Simultaneously, CI was used to target the cofilin pathway, offering a unique avenue to modulate microglial activation and inflammation induced by excess iron.

## 2 MATERIALS AND METHODS

### 2.1 Cell culture

The HMC‐3 line, distributed under the name of HMC‐3 Human Microglia Clone 3 (ATCC®CRL‐3304, RRID:CVCL_II76), was purchased from American Type Culture Collection (ATCC®) (Lot Number: 70043235). The cell line is not listed as a commonly misidentified cell line by the International Cell Line Authentication Committee (ICLAC). The certificate of analysis is provided in the Supporting Information. The HMC3 cells were cultured in Eagle's Minimum Essential Medium (EMEM) (ATCC®, Manassas, VA, USA, Cat. No. 30‐2003) supplemented with 10% heat‐inactivated fetal bovine serum (FBS) (ATCC® Manassas, VA, USA Cat. No.30‐2020), 2 mM L‐glutamine, 1 mM sodium pyruvate, and 1% penicillin/streptomycin (Gibco Cat. No. 15140122). All treatments, including FeSO_4_, DFX, and CI, were performed in the presence of 10% heat‐inactivated FBS. The culture was maintained at 37°C in a humidified atmosphere consisting of 95% air and 5% CO_2_.

### 2.2 Cell viability assay

HMC‐3 cells (below passage 10) were seeded in 96‐well plates. After reaching the desired confluency, the cells were exposed to various combinations of FeSO_4_ (100 to 300 μM), DFX (150 μM), or CI (5 μM) for 48 h. After treatment, 10 μL of Cell Counting Kit‐8 (CCK‐8) reagent (Dojindo, Kumamoto, Kyushu, Japan Cat. No. CK04‐11) was added to each well and incubated at 37°C for 2 h according to the manufacturer's protocol. The CCK‐8 reagent contains 2‐(2‐methoxy‐4‐nitrophenyl)‐3‐(4‐nitrophenyl)‐5‐(2,4‐disulfophenyl)‐2H tetrazolium, monosodium salt, which is enzymatically reduced by cellular dehydrogenases to produce an orange‐colored formazan product soluble in the culture medium. The quantity of formazan dye generated correlates directly with the number of viable cells. Absorbance was measured at 450 nm using a microplate reader, and the values were normalized to the control, representing a percentage change in cell viability or death.

### 2.3 Western blotting

For Western blotting (WB) cells were lysed by homogenizing them in ice‐cold RIPA buffer (Thermo Fisher Scientific, Waltham, MA, USA Cat. No. 89900) supplemented with a protease and phosphatase inhibitor cocktail (Sigma‐Aldrich, St. Louis, MO, USA, Cat. No. 78440) for 30 min. The supernatants containing the total protein fraction were collected following centrifugation at 14,000 × g for 15 min. The subcellular fractions were obtained using previously established protocols (Dimauro et al., 2012; Madineni et al., 2016). The protein concentration was determined using the Bradford reagent (Bio‐Rad Laboratories, Hercules, CA, USA, Cat. No. 5000006) per the manufacturer's instructions. Equal amounts of proteins from each sample were loaded onto 12% sodium dodecyl sulfate (SDS)‐polyacrylamide gels, separated by electrophoresis, and subsequently transferred to polyvinylidene fluoride (PVDF) membranes. These membranes were then blocked with 3% bovine serum albumin (BSA) (Sigma‐Aldrich, St. Louis, MO, USA, Cat. No. 9048‐46‐8) for 1 h to prevent non‐specific binding. Following this, the membranes were incubated overnight at 4°C with various primary antibodies, including ferritin heavy chain (FTH‐1) (Cell Signaling Technology, Danvers, MA, USA, Cat. No. 4393), ferritin light chain (FTL) (Proteintech Group, Inc., Cat no. 10727‐1‐AP), Divalent Metal Transporter 1 (DMT‐1) (Proteintech Group, Inc., Cat. No. 20507‐1‐AP), cofilin (Sigma‐Aldrich, St. Louis, MO, USA, Cat. No.5175), and phosphorylated cofilin (p‐cofilin) (Sigma‐Aldrich, St. Louis, MO, USA, Cat. No. 3313). For subcellular fractions, the primary antibodies were rabbit anti‐histone H3 (Cat no. 4499), rabbit anti‐nuclear factor kappa B (NF‐κB) p65 (Cat. No. 8242), and mouse anti‐β‐actin (Cat no. 4967) (1 : 1000). After washing with Tris‐buffered saline + Tween 20 (TBST) three times for 10 min each, the membranes were incubated with horseradish peroxidase‐conjugated goat anti‐mouse (Cat. No. 7076) and anti‐rabbit (Cat no. 7074) secondary antibodies (Cell Signaling Technology, Danvers, MA, USA) for 1 h at room temperature. β‐actin served as a loading control for cytosolic proteins and histone H3 for nuclear proteins. Finally, images were captured using the Syngene Imaging System (Frederick, MD, USA), and bands were analyzed using ImageJ software 1.53 t (National Institutes of Health, Bethesda, Maryland, USA).

### 2.4 Real‐time quantitative polymerase chain reaction

Total mRNA was extracted from the cells using TRIzol reagent (Invitrogen, Carlsbad, CA, USA, Cat. No. 15596018). Subsequently, complementary DNA (cDNA) was synthesized using the High‐Capacity cDNA Reverse Transcription Kit (Applied Biosystems™ Cat. No. 4368813). The mRNA expression levels were quantified using Power SYBR™ Green PCR Master Mix (Applied Biosystems™) in the StepOnePlus Real‐Time PCR System (Applied Biosystems™, Cat no. 4368702). β‐actin was utilized as a housekeeping control. The relative abundance of gene expression was determined using the double delta *Ct* method (2^−ΔΔ*Ct*^). For reverse transcription quantitative polymerase chain reaction (RT‐qPCR), the following primer pairs were employed: tumor necrosis factor‐alpha (TNF‐α): Forward‐ AGAACTCACTGGGGCCTACA, Reverse‐ AGGAAGGCCTAAGGTCCACT; IL‐6: Forward‐ GGTCCAGTTGCCTTCTCCC, Reverse‐ AGAGGTGAGTGGCTGTCTGT; IL12: Forward‐ CAGAAGGCCAGACAAACTCT, Reverse‐ GGTCTCTCTGGAATTTAGGCA; β‐actin: Forward‐ GATTCCTATGTGGGCGACGA, Reverse TGTAGAAGGTGTGGTGCCAG; interleukin 1 beta (IL‐1β): Forward‐ TCGCCAGTGAAATGATGGCT, Reverse‐GGTCGGAGATTCGTAGCTGG.

### 2.5 ELISA assay

HMC‐3 cells were seeded in a 6‐well plate and, after reaching the desired confluency, treated with FeSO_4_ (100 μM), DFX (150 μM), and CI (5 μM) for 48 h. Following treatment, the supernatant was collected, and the concentrations of TNF‐α, IL‐6, interleukin 12 (IL‐12), and IL‐1β were quantified using enzyme‐linked immunosorbent assay (ELISA) kits following the manufacturer's instructions (R&D Systems Inc., Minneapolis, MN Cat. No's. DY206, DY201, DY210, DY202). The levels of inflammatory cytokines were determined by comparing the absorbance values to a standard curve generated from known concentrations of the respective inflammatory cytokines. The results were then expressed in pg/mL of medium.

### 2.6 Immunocytochemistry

The immunocytochemistry was performed using established protocols (Marchenko & Flanagan, 2007). The HMC‐3 cells were cultured in EMEM (supplemented with 10% FBS and 1% Penstrep) on poly‐D‐lysine (PDL) (Gibco Cat no. A3890401) coated coverslips in a 24‐well plate for 24 h or until the desired confluency was achieved. Subsequently, the cells were treated for 48 h with different combinations of FeSO_4_, DFX, or CI, and an untreated control group. Following treatment, cells were briefly washed with 1X phosphate‐buffered saline (PBS) (pH 7.4), fixed using 4% paraformaldehyde, and then permeabilized by 0.25% Triton X‐100 (Sigma‐Aldrich, St. Louis, MO, USA, Cat. No. 50‐165‐7277). After fixation and permeabilization, cells were incubated in 1% BSA and 22.52 mg/mL glycine in PBST (PBS+ 0.1% Tween 20) for 30 min to block non‐specific binding of the antibodies. After blocking, the cells were incubated with primary antibodies, rabbit anti‐cofilin (1 : 400; Cell Signaling Technology, Danvers, MA, USA), and separately rabbit NFκB (1 : 250; Cell Signaling Technology, Danvers, MA, USA) overnight at 4°C with gentle rocking. Subsequently, Alexa Fluor® 647 AffiniPure™ Fab Fragment Goat Anti‐Rabbit IgG (H + L) secondary antibody (RED) (1 : 1000; Jackson ImmunoResearch Laboratories INC, RRID:AB_2338084) and Anti‐rabbit IgG (H + L), F(ab′)2 Fragment (Alexa Fluor® 488 Conjugate) secondary IgG antibody (GREEN) (1 : 1000; Cell Signaling Technology, Danvers, MA, USA, Cat No. 4412) were applied at room temperature for 1 h for cofilin and NFκB, respectively. After three PBS rinses (5 min each), coverslips were mounted with Fluoromount‐G™, with DAPI (Invitrogen™, Cat. No. 00‐4959‐52) on glass slides, and the gaps were sealed. Cell imaging was performed using fluorescent microscopy at 40× magnification.

### 2.7 Statistical analysis

The experimental results were expressed as the mean ± standard error of the mean (SEM) and are accompanied by the number of observations (independent preparations of cultured cells). Data were analyzed by one‐way ANOVA followed by post hoc Tukey's multiple comparisons test using Graph Pad Prism 9.0.0 (GraphPad Software, San Diego, CA). A value of *p* < 0.05 is considered to be statistically significant. Normality was confirmed using the Shapiro–Wilk test. No test for outliers was conducted.

## 3 RESULTS

### 3.1 FeSO_4_ reduces the cell viability of HMC‐3 microglia

**FIGURE 1:**
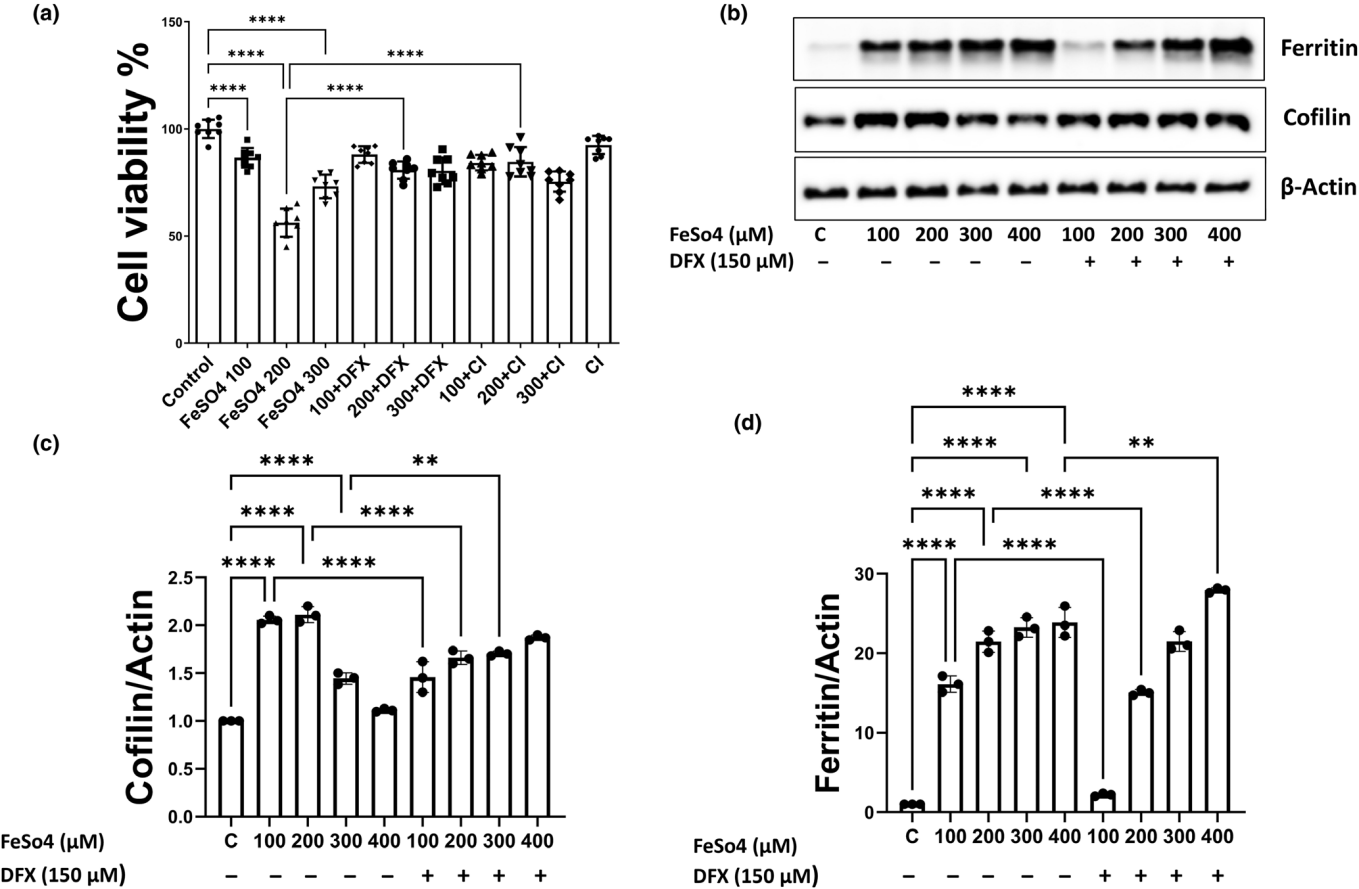
Cell viability protein expression of ferritin and cofilin in HMC‐3 cells upon exposure to various concentrations and combinations of FeSO_4_, DFX, and CI. (a) A decrease in viability of HMC‐3 cells after challenging with different concentrations of FeSO_4_ was observed. Both DFX and CI restored the cell viability significantly. Data show mean ± SEM, *n* = 8 cell culture preparations. Normality of residuals was confirmed using Shapiro–Wilk test (*p* > 0.05). Statistical analysis was performed using one‐way ANOVA (*F*(10, 77) = 40.26, *p* < 0.0001, *R* ^2^ = 0.8394) with Tukey's multiple comparisons test (*p* < 0.05) for post hoc analysis. Significant comparisons: (**p < 0.01; ****p < 0.0001). (b) Representative blots of protein expression of ferritin, cofilin, and β‐actin (loading control) with different concentrations of FeSO_4_ and DFX. FeSO_4_ (100 μM) resulted in a significant over‐expression of cofilin and DFX (150 μM) effectively mitigating the effect in HMC‐3 cells exposed to different concentrations of FeSO_4_. (c) WB analysis of cofilin expression in HMC‐3 cells with different concentrations of FeSO_4_ and DFX 150 μM for 48 h. FeSO_4_ (100 μM) resulted in a significant over‐expression of cofilin and DFX (150 μM) effectively mitigating the effect. Statistical analysis was performed using two‐tailed one‐way ANOVA followed by Tukey's multiple comparisons test (*F*(8, 18) = 91.13, *p* < 0.0001). Data are presented as mean ± SEM (*n* = 3). Normality of the data was confirmed using the Shapiro–Wilk test (*W* = 0.9247, *p* = 0.0514). (d) WB  analysis of ferritin expression in HMC‐3 cells after exposure with different concentrations of FeSO_4_ and DFX 150 μM for 48 h. FeSO_4_ (100 μM) resulted in a significant over‐expression of ferritin and DFX (150 μM) effectively mitigating the effect. Statistical analysis was performed using two‐tailed one‐way ANOVA followed by Tukey's multiple comparisons test (*F*(8, 18) = 249.8, *p* < 0.0001). Data are presented as mean ± SEM (*n* = 3). Normality of the data was confirmed using the Shapiro–Wilk test (*W* = 0.9451, *p* = 0.1631) (**p < 0.01 and ****p < 0.0001) (CI, cofilin inhibitor; DFX, deferoxamine; FeSO_4_, ferrous sulfate).

The HMC‐3 cells were exposed to various combinations of FeSO_4_ (100 to 300 μM), DFX (150 μM), or CI (5 μM) for 48 h. Subsequently, 10 μL of CCK‐8 reagent was added to each well and incubated at 37°C for 2 h. The results demonstrated that FeSO_4_ exposure reduced the viability of HMC‐3 cells in all concentrations. Thereafter, we selected 100 μM concentration of FeSO_4_ for the rest of our experiments as this concentration did not show any drastic reduction in cell viability (Figure 1a).

### 3.2 FeSO_4_ exposure increases the ferritin and cofilin expressions in HMC‐3 cells

To investigate cofilin and ferritin expressions in microglia after FeSO_4_ exposure, HMC‐3 cells were exposed to varying concentrations of FeSO_4_ (100, 200, 300, and 400 μM) and co‐treated with DFX (150 μM) for 48 h. A significant increase in ferritin expression with 100 μM FeSO_4_ was mitigated by 150 μM of DFX. Cofilin expression also increased significantly with 100 μM FeSO_4_, which was mitigated by 150 μM DFX (Figure 1b–d). Subsequently, 100 μM FeSO_4_ and 150 μM DFX concentrations were chosen for the rest of the experiments.

### 3.3 Temporal expressions of cofilin, FTH, FTL, and DMT‐1 in HMC‐3 cells after exposure to FeSO_4_

**FIGURE 2:**
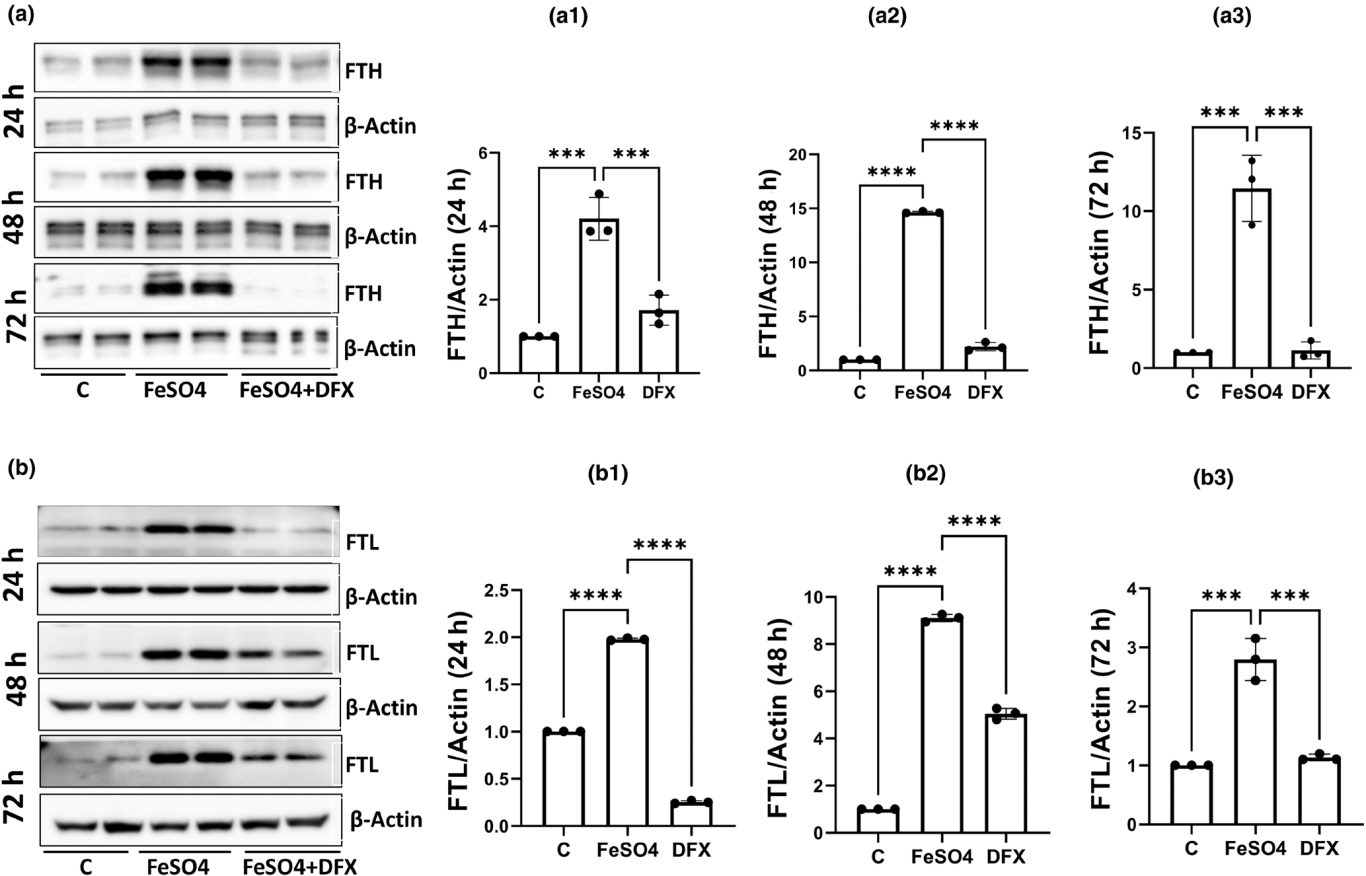
Temporal expression of FTH and FTL. (a, b) Representative blots of protein expression of FTH, FTL, and β‐actin (loading control) with FeSO_4_ and DFX treatment for different time periods (24, 48, and 72 h). (a1–a3 and b1–b3) WB analysis of FTH and FTL expressions in HMC‐3 cells. A significant over‐expression of both FTH and FTL was observed at all the time points and DFX mitigated these effects. The membrane was initially probed for β‐actin as a loading control. Subsequently, it was stripped and reprobed for FTL. The identical β‐actin bands between Figures 2b and 3b are because of the use of the same membrane, s.wiley.com/asset/photos/eletroni_artworktripped and reprobed for DMT‐1 after probing for FTL. For FTH‐1, statistical analysis was performed using two‐tailed one‐way ANOVA followed by Tukey's multiple comparisons test (*F*(2, 6) = 50.24, *p* < 0.0001 for 24 h; *F*(2, 6) = 3349, *p* < 0.0001 for 48 h; *F*(2, 6) = 67.95, *p* < 0.0001 for 72 h). Data are presented as mean ± SEM (*n* = 3). Normality of the data was confirmed using the Shapiro–Wilk test for all time points. For FTL, statistical analysis was performed using two‐tailed one‐way ANOVA followed by Tukey's multiple comparisons test (*F*(2, 6) = 17 775, *p* < 0.0001 for 24 h; *F*(2, 6) = 1912, *p* < 0.0001 for 48 h; *F*(2, 6) = 68.98, *p* < 0.0001 for 72 h). Data are presented as mean ± SEM (*n* = 3). Normality of the data was confirmed using the Shapiro–Wilk test or D'Agostino‐Pearson omnibus (K2) (***p < 0.001 and ****p < 0.0001) (CI, cofilin inhibitor; DFX, deferoxamine; FeSO_4_, ferrous sulfate; FTH, ferritin heavy chain; FTL, ferritin light chain).

**FIGURE 3:**
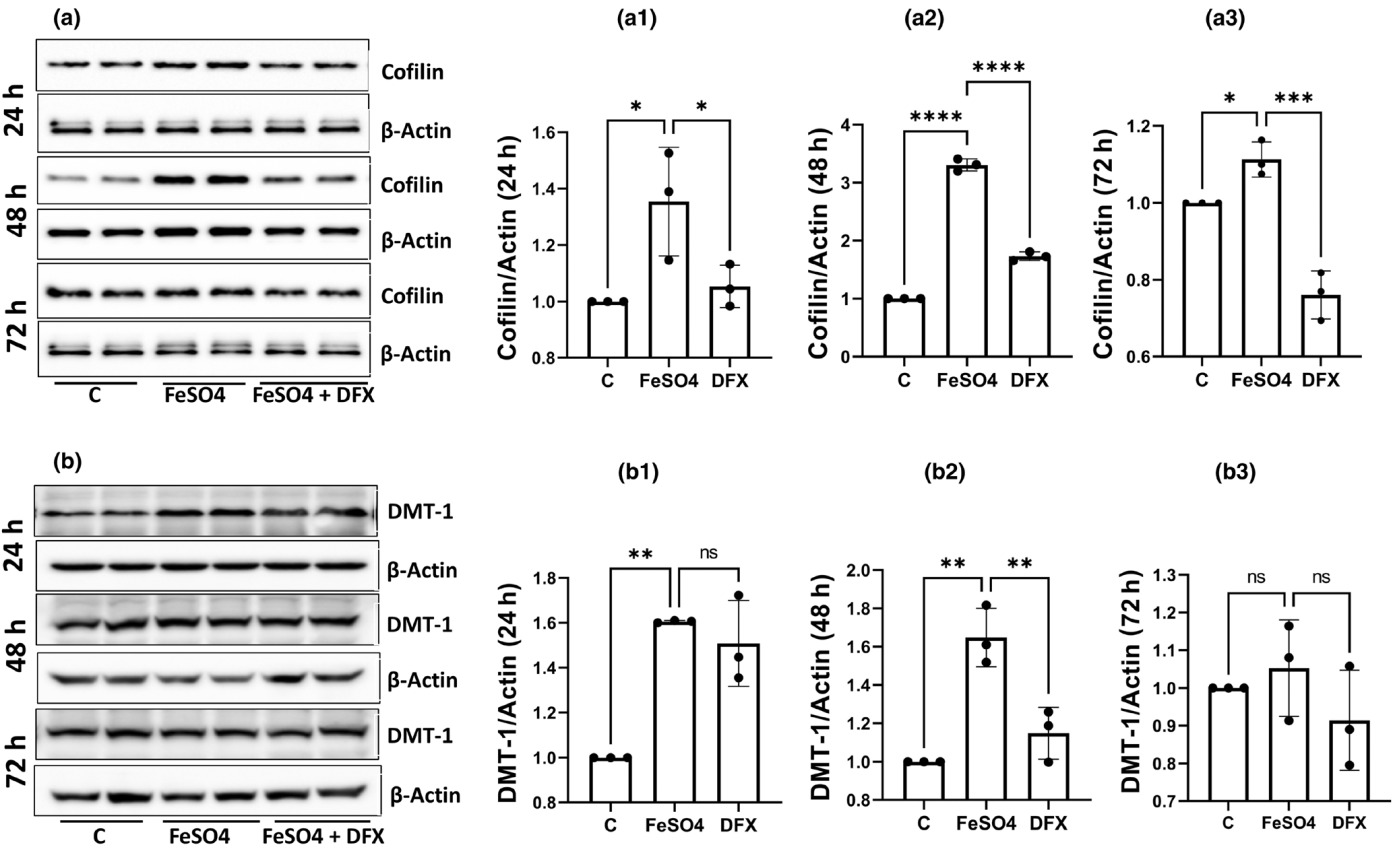
Temporal expression of cofilin and DMT‐1. (a, b) Representative blots of protein expression of Cofilin, DMT‐1, and β‐actin (loading control) with FeSO_4_ and DFX treatment for different time periods (24, 48, and 72 h). (a1–a3 and b1–b3) WB analysis of cofilin and DMT‐1 expressions in HMC‐3 cells. A significant over‐expression of cofilin was observed at all the time points and DFX mitigated these effects. DMT‐1 expression significantly increases with FeSO_4_ treatment at 24 and 48 h; however, on 48 h, treatment with DFX showed a significant decrease in DMT‐1 expression. The membrane was initially probed for β‐actin as a loading control, stripped, and reprobed for DMT‐1. The β‐actin bands are identical to those in Figure 2b, as the same membrane was used for probing both FTL and DMT‐1. For cofilin, statistical analysis was performed using two‐tailed one‐way ANOVA followed by Tukey's multiple comparisons test (*F*(2, 6) = 7.688, *p* = 0.0221 for 24 h; *F*(2, 6) = 760.7, *p* < 0.0001 for 48 h; *F*(2, 6) = 48.60, *p* = 0.0002 for 72 h). Data are presented as mean ± SEM (*n* = 3). Normality of the data was confirmed using the Shapiro–Wilk test for all time points. For DMT‐1, statistical analysis was performed using two‐tailed one‐way ANOVA followed by Tukey's multiple comparisons test (*F*(2, 6) = 23.23, *p* = 0.0015 for 24 h; *F*(2, 6) = 24.87, *p* = 0.0012 for 48 h; *F*(2, 6) = 1.286, *p* = 0.3428 for 72 h). Data are presented as mean ± SEM (*n* = 3). Normality of the data was confirmed using the Shapiro–Wilk test (*p < 0.05; **p < 0.01; ***p < 0.001 and ****p < 0.0001) (CI, cofilin inhibitor; DFX, deferoxamine; DMT‐1, divalent metal transporter 1; FeSO_4_, ferrous sulfate).

Following a 48‐h exposure to 100 μM FeSO_4_, HMC‐3 microglia exhibited the highest increase in cofilin expression and co‐treatment with 150 μM DFX mitigated this effect. Western blot analysis at different time points (24, 48, and 72 h) confirmed a consistent increase in FTH, FTL (Figure 2a,b), and cofilin expression (Figure 3a) in FeSO_4_‐exposed cells across all time points. Co‐treatment with DFX significantly reduced the expressions of ferritin (FTH and FTL) and cofilin compared to the FeSO_4_ group. Notably, the most prominent increase in cofilin expression was observed at the 48‐h time, which was selected for subsequent experiments (Figure 3a). FeSO_4_ exposure for 24 h resulted in a significant increase in DMT‐1 expression, and although DFX co‐treatment reduced DMT‐1 expression, the decrease was not statistically significant. At 48 h, DMT‐1 expression was significantly increased in the FeSO_4_ group, and DFX co‐treatment mitigated this effect. No significant changes in DMT‐1 expression were observed at the 72 h time point (Figure 3b).

### 3.4 CI mitigates the FeSO_4_ ‐induced cofilin expression in HMC‐3 cells

**FIGURE 4:**
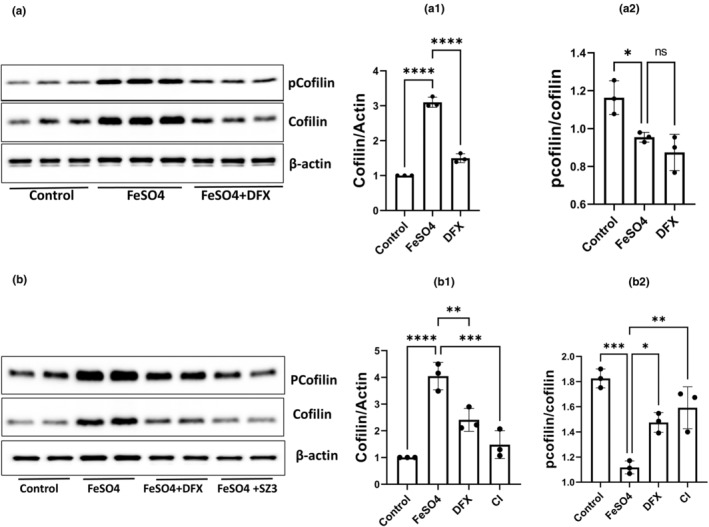
CI mitigates the FeSO_4_‐induced cofilin expression (a, b) WB showing expression of cofilin and phosphocofilin in HMC‐3 cells in response of exposure to FeSO_4_ and treatment with DFX and CI for 48 h (β‐actin is loading control). (a1, b1) Quantitative analysis of cofilin protein expression shows a significant over‐expression of cofilin in FeSO_4_ group and the effect being mitigated by DFX and CI. (a2, b2) Ratio of pcofilin/cofilin protein expression in HMC‐3 cells with FeSO_4_ exposure and DFX and CI treatments. A significant decrease was observed in pcofilin/cofilin ratio in the FeSO_4_ group. Data show mean ± SEM, *n* = 3 cell culture preparations. One‐way ANOVA test with Tukey's correction for multiple comparisons was performed (*p < 0.05; **p < 0.01; ***p < 0.001 and ****p < 0.0001) (CI, cofilin inhibitor; DFX, deferoxamine; FeSO_4_, ferrous sulfate).

**FIGURE 5:**
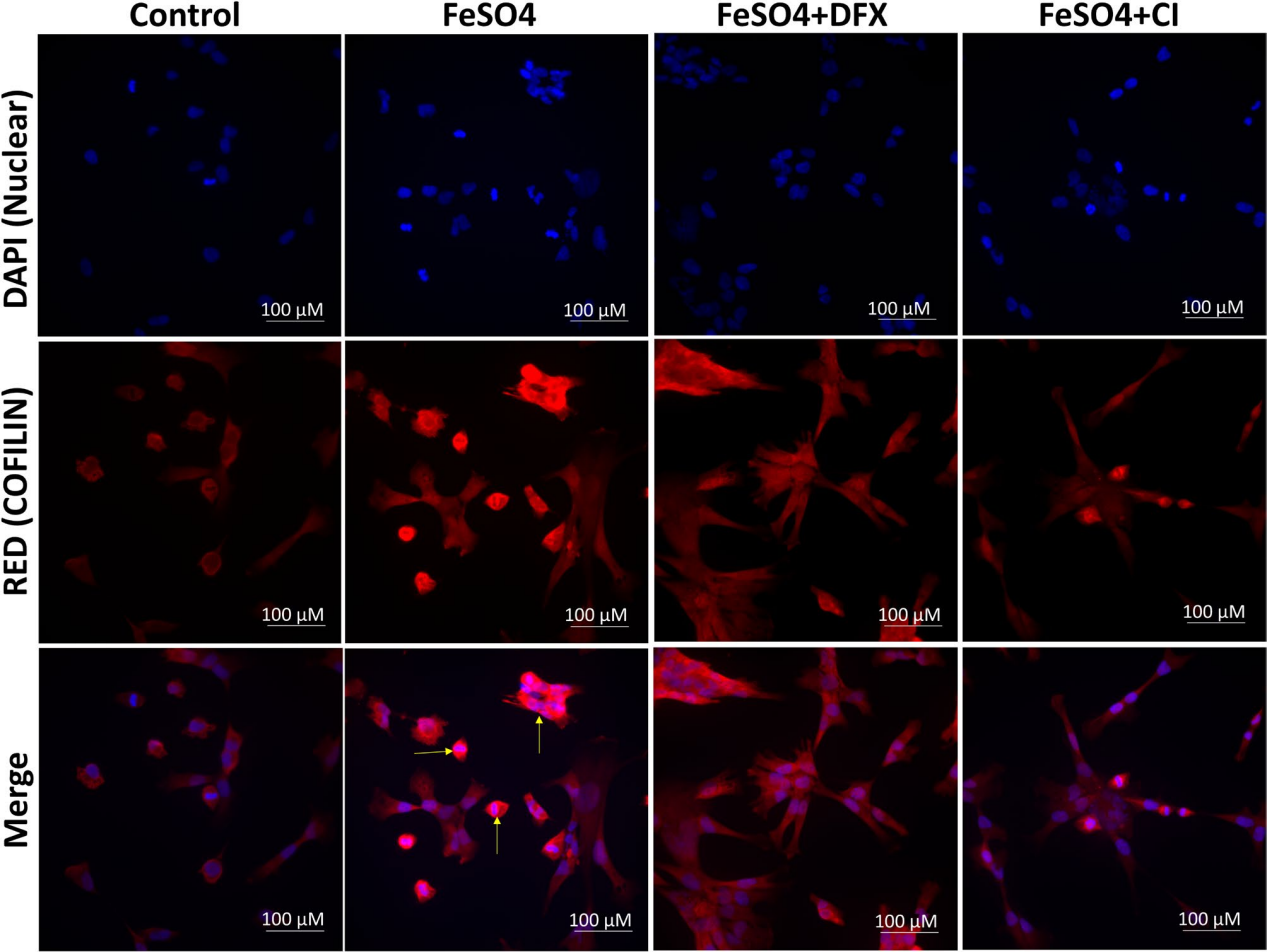
Cofilin over‐expression in HMC‐3 cells after FeSO_4_ exposure. Cofilin expression in response to FeSO_4_ exposure and treatment with DFX and CI was visualized by immunocytochemistry using cofilin antibody (Texas red) with nuclear stain DAPI (Blue). Representative immunofluorescence pictures show increased fluorescence of cofilin (stained red) in FeSO_4_ group (yellow arrows) as compared with the control group. Further, there was a decrease in the red fluorescence (cofilin) in DFX and CI groups as compared with the FeSO_4_ group (CI, cofilin inhibitor; DFX, deferoxamine; FeSO_4_, ferrous sulfate).

Next, we analyzed the expressions of cofilin and phosphocofilin in HMC‐3 cells following the exposure to 100 μM FeSO_4_ and co‐treatment with DFX 150 μM for 48 h. There was a significant increase in cofilin expression in FeSO_4_‐exposed group and a decrease in DFX‐treated group. Interestingly, phosphocofilin levels followed a similar trend; however, cofilin/phosphocofilin ratio was higher in the FeSO_4_ group. Subsequently, we investigated the impact of CI on cofilin expression in FeSO_4_‐exposed microglia. HMC‐3 cells exposed to 100 μM FeSO_4_ and treated separately with 150 μM DFX, and 5 μM CI for 48 h showed an increase in cofilin expression in the FeSO_4_‐treated group; however, co‐treatment with DFX or CI significantly reduced cofilin expression compared to the FeSO_4_ group (Figure 4a,b). To further validate our results, we performed immunocytochemistry using a similar experimental design. The results revealed that the FeSO_4_‐exposed group exhibited an increased fluorescence of cofilin (stained red) compared to the control group. In contrast, both the DFX and CI groups displayed a reduction in the red fluorescence (corresponding to cofilin expression) compared to the FeSO_4_ group (Figure 5). These findings suggest that the treatment with DFX or CI decreased the cofilin expression induced by FeSO_4_ exposure in HMC‐3 microglia. So, it could be concluded that FeSO_4_ activated cofilin, and the effect can be mitigated by DFX or CI treatment.

### 3.5 CI inhibits FeSO_4_ ‐induced inflammatory cytokine production in HMC3 cells

**FIGURE 6:**
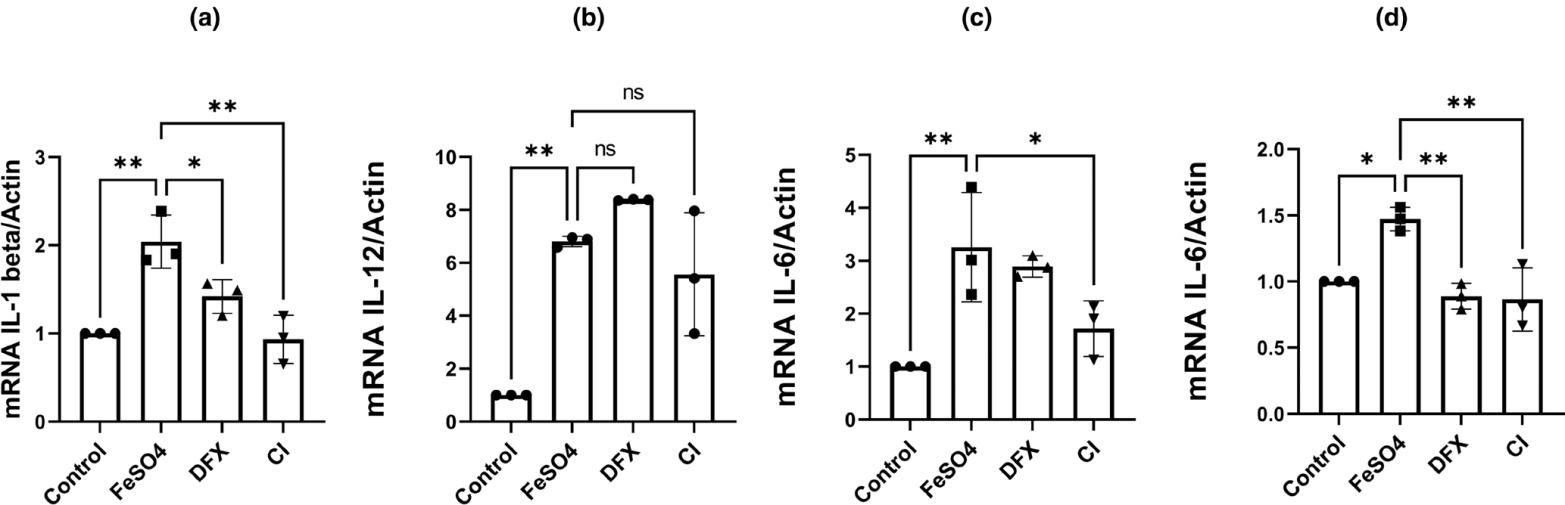
Up‐regulation of mRNA levels in HMC‐3 cells exposed to FeSO_4_. The mRNA levels of various inflammatory cytokines were detected by real‐time PCR. (a) Increased TNF‐α mRNA with FeSO_4_ exposure, while both DFX and CI mitigated this effect. Statistical analysis was performed using one‐way ANOVA followed by Tukey's multiple comparisons test (*F*(3, 8) = 9.440, *p* = 0.0053). Data are presented as mean ± SEM (*n* = 3). Normality was confirmed using the Shapiro–Wilk test. (b) The FeSO_4_ exposure results in a significant increase in the mRNA expression of IL‐12; however, neither DFX nor CI had any effect on this change. Statistical analysis was performed using one‐way ANOVA followed by Tukey's multiple comparisons test (*F*(3, 8) = 22.32, *p* = 0.0003). Data are presented as mean ± SEM (*n* = 3). Normality was not met as indicated by the Shapiro–Wilk test. (c) There was a significant increase in mRNA expression of IL‐1β with FeSO_4_ exposure, and the effect was mitigated with the co‐treatment of either DFX or CI. Statistical analysis was performed using one‐way ANOVA followed by Tukey's multiple comparisons test (*F*(3, 8) = 15.48, *p* = 0.0011). Data are presented as mean ± SEM (*n* = 3). Normality was confirmed using the Shapiro–Wilk test. (d) FeSO_4_ exposure increased the IL‐6 mRNA levels, and the effect was mitigated by both DFX and CI. Data show mean ± SEM, *n* = 3 cell culture preparations. Statistical analysis was performed using one‐way ANOVA followed by Tukey's multiple comparisons test (*F*(3, 8) = 12.90, *p* = 0.0020). Data are presented as mean ± SEM (*n* = 3). Normality was confirmed using the Shapiro–Wilk test (ns, non‐significant; *p < 0.05 and **p < 0.01) (CI, cofilin inhibitor; DFX, deferoxamine; FeSO_4_, ferrous sulfate; IL‐12, interleukin 12; IL‐1β, interleukin 1 beta; IL‐6, interleukin 6; TNF‐α, tumor necrosis factor‐alpha).

**FIGURE 7:**
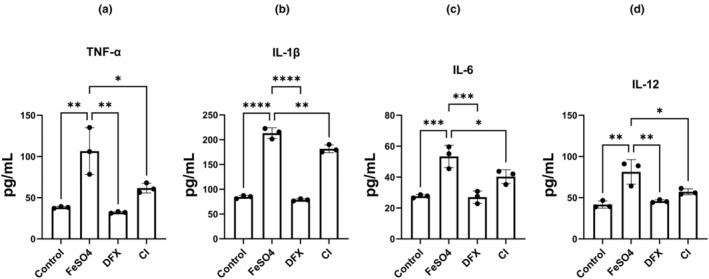
Cofilin inhibitor suppresses the inflammatory cytokine production in HMC‐3 cells exposed to FeSO_4_. The levels of various inflammatory cytokines were measured by ELISA. (a–d) ELISA quantification of various inflammatory cytokines released into the media by HMC‐3 cells upon exposure to FeSO_4_ and treatment with DFX and CI. The levels of all the four cytokines (TNF‐α, IL‐12, IL‐1β, and IL‐6) measured showed significant increase in the FeSO_4_ group. Similarly, co‐treatments with either DFX or CI significantly reduced the production of these cytokines Data show mean ± SEM, *n* = 3 cell culture preparations. One‐way ANOVA test with Tukey's correction for multiple comparisons was performed (TNF‐α—(*F*(3, 8) = 16.19, *p* = 0.0009); IL‐1β—(*F*(3, 8) = 289.7, *p* < 0.0001); IL‐6—(*F*(3, 8) = 20.92, *p* = 0.0004); IL‐12—(*F*(3, 8) = 14.77, *p* = 0.0013)). Normality was confirmed using the Shapiro–Wilk test (*p < 0.05; **p < 0.01; ***p < 0.001 and ****p < 0.0001) (CI, cofilin inhibitor; DFX, deferoxamine; FeSO_4_, ferrous sulfate; IL‐12, interleukin 12; IL‐1β, interleukin 1 beta; IL‐6, interleukin 6; TNF‐α, tumor necrosis factor‐alpha).

FeSO_4_ treatment resulted in an elevation of mRNA expression of several inflammatory cytokines in HMC‐3 cells. After 48 h of exposure to FeSO_4_ and treatment with DFX, and CI, the FeSO_4_‐treated group showed a significant increase in the mRNA expression of TNF‐α, IL‐12, IL‐1β, and IL‐6 (Figure 6a–d). There was a significant decrease in mRNA expression of TNF‐α, IL‐1β, and IL‐6 (a, c, and d) in response to CI treatment. DFX treatment significantly decreased the mRNA expressions of IL‐1β and IL‐6; however, the effect on TNF‐α was not statistically significant (Figure 6a). Moreover, FeSO_4_ treatment resulted in a significant increase in IL‐12 mRNA expression; however, neither DFX nor CI could significantly decrease IL‐12 mRNA expression (Figure 6b). We also performed the ELISA to check for inflammatory cytokine production in the groups mentioned above. The FeSO_4_‐treated group exhibited a significant increase in TNF‐α, IL‐1β, IL‐12, and IL‐6 expressions. In contrast, the groups treated with DFX or CI demonstrated mitigation of these inflammatory effects (Figure 7a–d).

### 3.6 CI inhibits FeSO_4_ ‐induced NF‐κB over‐expression

**FIGURE 8:**
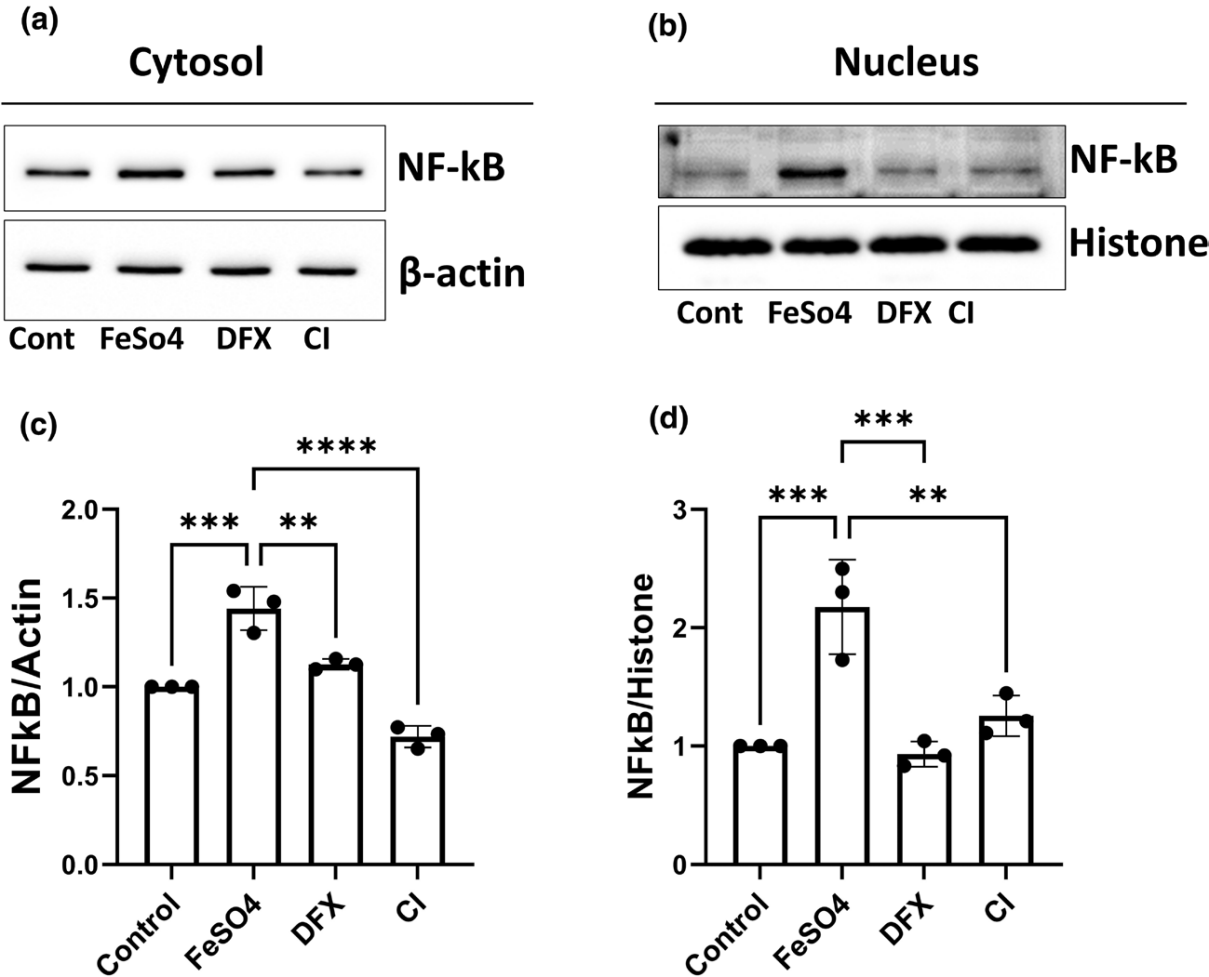
Over‐expression of NF‐kB in FeSO_4_‐exposed HMC‐3 cells. WB of the cytosolic and nuclear fractions was performed for NF‐kB protein expression. (a, b) WB showing over‐expression of NF‐kB in cytosolic and nuclear fractions of HMC‐3 cells in response to FeSO_4_ exposure and treatment with DFX and CI mitigated the effect (β‐actin and histone served as loading controls for cytosolic and nuclear fractions, respectively). (c, d) Quantitative analysis of NF‐kB protein expression in the cytosolic and nuclear fractions in HMC‐3 cells in response to FeSO_4_ exposure and treatment with DFX and CI. There is a significant increase in the expression of NF‐kB with FeSO_4_ exposure in both cytosolic and nuclear fractions of HMC‐3 cells. The DFX or CI co‐treatment effectively inhibits the over‐expression of NF‐kB in both the fractions. Data show mean ± SEM, *n* = 3 cell culture preparations. One‐way ANOVA test with Tukey's correction for multiple comparisons was performed, cytosolic—(*F*(3, 8) = 54.82, *p* < 0.0001); nuclear—(*F*(3, 8) = 19.74, *p* = 0.0005). Data are presented as mean ± SEM (*n* = 3). Data are presented as mean ± SEM (*n* = 3). Normality was confirmed using the Shapiro–Wilk test (**p < 0.01; ***p < 0.001 and ****p < 0.0001) (CI, cofilin inhibitor; DFX, deferoxamine; FeSO_4_, ferrous sulfate; NF‐κB, nuclear factor kappa B).

**FIGURE 9:**
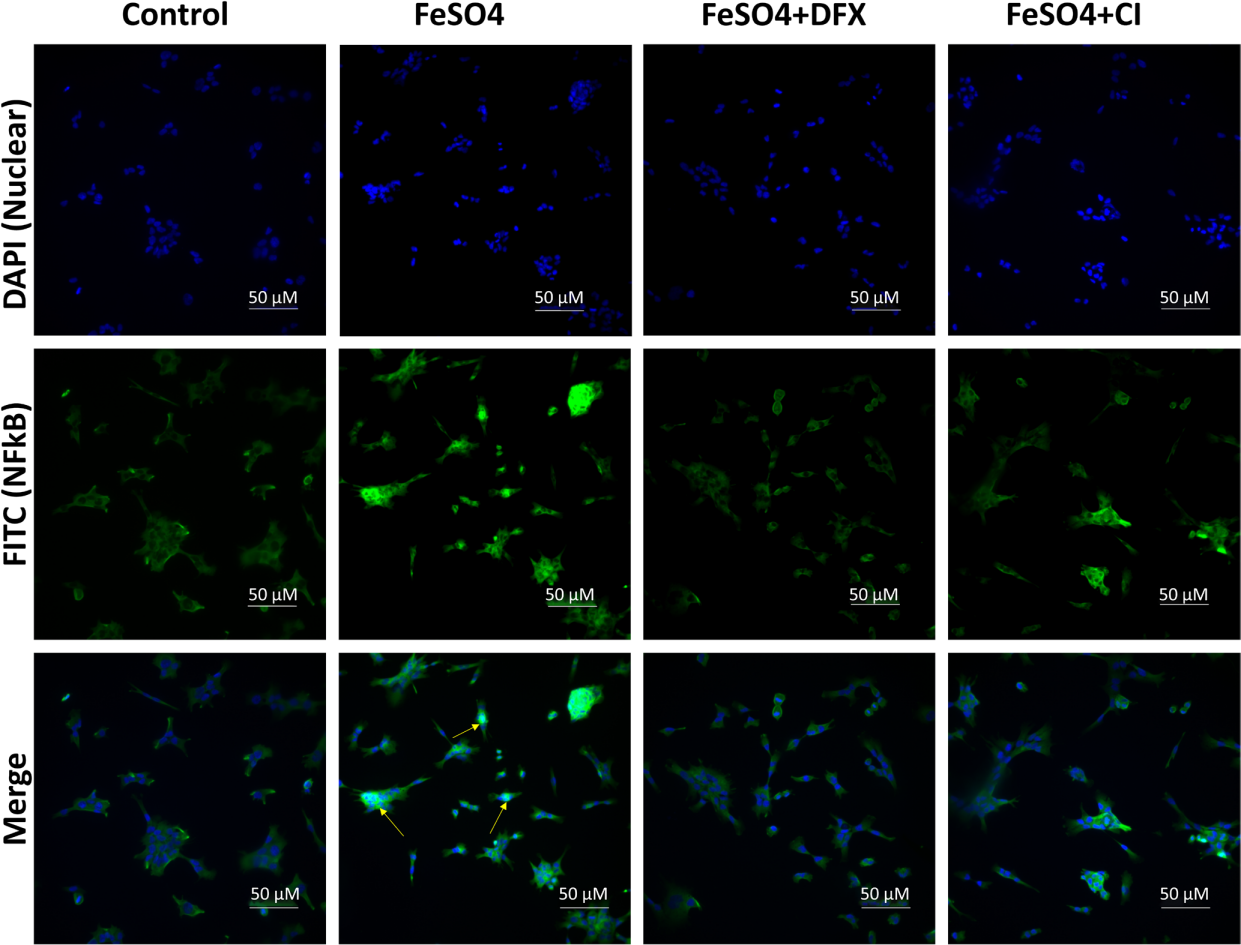
FeSO_4_induced NF‐kB over‐expression. NFkB expression in response to FeSO_4_ exposure and treatments with DFX and CI was visualized by immunocytochemistry for NFkB antibody (FITC green) with nuclear stain DAPI (Blue). Representative immunofluorescence pictures show increased fluorescence of NFkB in the nuclear region (stained green) in FeSO_4_ group (Yellow arrows) as compared with the control group. Furthermore, there was a decrease in the fluorescence (NFkB/Green) in DFX and CI groups as compared with the FeSO_4_ group (CI, cofilin inhibitor; DFX, deferoxamine; FeSO_4_, ferrous sulfate; NF‐κB, nuclear factor kappa B).

HMC‐3 cells harvested after different treatments were subjected to subcellular fractionation to isolate cytosolic and nuclear fractions; later, WB was performed to check the protein expression of NF‐κB. A significant over‐expression of NF‐κB was observed in the FeSO_4_‐exposed cells compared to the control in both subcellular fractions. Interestingly, both the DFX and CI groups exhibited a reduction in NF‐κB expression in both fractions (Figure 8a,b). Notably, the reduction was more pronounced in the nuclear fraction (Figure 8b). These results suggest that DFX and CI effectively inhibit NF‐κB activation in microglia. To further validate our results, we performed the immunocytochemistry of HMC‐3 cells with similar experimental conditions. After 48 h of treatment, the immunocytochemistry was performed using NF‐κB antibody (FITC green) and nuclear stain DAPI (Blue). The results indicated that FeSO_4_ exposure exhibited an increased intensity of green fluorescence (corresponding to NF‐κB expression) in the nuclear region compared to the control. Conversely, both the DFX and CI groups displayed a decrease in NF‐κB fluorescence (Green) compared to the FeSO_4_ exposed cells (Figure 9). These findings suggest that the treatment of DFX and CI may have a mitigating effect on the elevated NF‐κB expression induced by FeSO_4_ in HMC‐3 microglia.

## 4 DISCUSSION

In this study, we observed a strong association between iron dysregulation and inflammatory responses in HMC‐3 cells. Upon exposure to high iron concentration, the microglia (HMC‐3) displayed up‐regulated cofilin expression and increased levels of different inflammatory cytokines. The iron overload in HMC‐3 cells was confirmed by the up‐regulated expression of iron storage protein, ferritin. Since FeSO_4_ is transported via DMT‐1 into the cells, DMT‐1 expression was also evaluated and found to be up‐regulated at certain time points upon FeSO_4_ exposure. Moreover, the inflammatory effects were mitigated by either CI or DFX, suggesting an important role of cofilin in the inflammatory cascade induced by iron overload in microglia and a potential therapeutic effect of DFX and CI.

Iron dysregulation has been implicated in various neurological disorders (Kenkhuis et al., 2022), and our study delves into the specific impact of excess iron on microglia, shedding light on the intricate relationship between iron homeostasis, cofilin expression, and microglia activation in the context of neuroinflammation. Our results demonstrate a significant increase in cofilin expression by microglia following treatment with excess iron (FeSO_4_). Cofilin, a vital regulator of actin dynamics, is pivotal in cellular processes such as migration, phagocytosis, and cytokine release (Samstag et al., 2013). The observed up‐regulation of cofilin in response to excess iron suggests its involvement in iron‐induced microglial activation. The most likely mechanism for iron‐induced cofilin expression involves the activation of the NF‐κB signaling pathway and the accompanying inflammatory response, as well as the need for cytoskeletal remodeling under iron‐induced stress conditions. We propose that the increase in cofilin observed in our study is driven by these combined processes. These findings align with emerging evidence highlighting cofilin and iron as key mediators in neuroinflammation and neurodegeneration (Alsegiani & Shah, 2020; Lapeña‐Luzón et al., 2021; Ndayisaba et al., 2019; Ward et al., 2022; Yan et al., 2022).

The cofilin/phospho‐cofilin ratio, a marker of cofilin activity, revealed a pronounced increase in cofilin levels with FeSO_4_ treatment. This suggests an active involvement of cofilin in the cellular response to excess iron, possibly contributing to microglial pro‐inflammatory phenotypes. Previous studies have linked cofilin activation to inflammatory responses, indicating its potential as a therapeutic target in iron‐associated neuroinflammatory conditions (Donley et al., 2021; Minamide et al., 2000; van Rheenen et al. 2009; Wang et al., 2020). To explore potential therapeutic avenues, we employed a commonly used iron chelator DFX and a newly synthesized CI from our lab (Alaqel et al., 2022). DFX, known for sequestering excess iron, mitigated iron‐induced cofilin expression in microglia. This highlights the importance of iron chelation as a potential therapeutic strategy to counteract the detrimental effects of excess iron in neuroinflammation. Furthermore, CI exhibited a significant reduction in cofilin expression, suggesting its efficacy in inhibiting cofilin activation. These findings underscore the therapeutic potential of targeting cofilin to mitigate iron‐induced microglial activation, providing a novel avenue for intervention in neuroinflammatory conditions associated with iron dysregulation.

Our study also investigated the downstream inflammatory consequences of excess iron on microglia. FeSO_4_ treatment significantly increased the mRNA expression of inflammatory cytokines such as TNF‐α, IL‐1β, and IL‐6. Our results are in agreement with the previous research that shows increased expression of various inflammatory cytokines by microglia associated with iron up‐regulation (Andersen et al., 2014; Rathnasamy et al., 2011; Riederer et al., 2023). Consistently, ELISA results confirmed elevated cytokine levels in the FeSO_4_‐treated group. Importantly, both DFX and CI demonstrated a mitigating effect on the expression of these inflammatory cytokines. These findings emphasize the intricate link between excess iron and the inflammatory milieu within microglia. The ability of DFX and CI to alleviate inflammatory cytokine expression further supports their potential as therapeutic agents in iron‐associated neuroinflammatory conditions. Our results on DFX further validate previous studies showing iron chelators such as DFX have preventive roles in neuroinflammatory diseases (Jia et al., 2023; Li et al., 2022).

In addition, our results show that CI treatment is equally good in suppressing the inflammatory cascade. Moreover, our investigation into the effects of excess iron and therapeutic interventions on NF‐κB activation further elucidates the molecular mechanisms underlying neuroinflammation. NF‐κB is a key transcription factor regulating inflammatory gene expression (Yu et al., 2020). Our results from subcellular fractionation revealed a significant over‐expression of NF‐κB in the FeSO_4_‐treated group, consistent with the observed inflammatory responses. Both DFX and CI exhibited a reduction in NF‐κB expression, particularly in the nuclear fraction. This suggests that DFX and CI effectively inhibit NF‐κB activation in microglia, highlighting their broader anti‐inflammatory effects. The observed reduction in NF‐κB expression following treatment with DFX and CI underscores their potential as therapeutic agents for mitigating neuroinflammation. By inhibiting NF‐κB activation, these agents may exert neuroprotective effects by attenuating the production of inflammatory mediators and mitigating the detrimental effects of neuroinflammation on neuronal function.

Taken together, our results demonstrate that excess iron results in the activation of NF‐κB in microglia, which subsequently activates the downstream pro‐inflammatory cascade and the DFX and CI possibly suppress the NF‐κB activation and ultimately decrease pro‐inflammatory effects. These results are in agreement with the previous investigations on the role of DFX in NF‐κB suppression and neuroprotection (Messa et al., 2008). Therapeutically targeting iron dysregulation is a promising avenue for mitigating neuroinflammation in various neurological disorders. Our study demonstrates the efficacy of both DFX and our novel CI in attenuating the effects of excess iron on microglia, suggesting a possible link between iron‐induced cofilin activation and pro‐inflammatory phenotype of microglia. Given the association between iron overload, neuroinflammation, and various neurological disorders, including neurodegenerative diseases such as AD, PD, and ischemic stroke, understanding the role of excess iron in microglial activation becomes crucial (Aljuhani et al., 2020; Gao et al., 2023; Kenkhuis et al., 2021; Ndayisaba et al., 2019; Thomsen et al., 2015; Vela, 2018; Ward et al., 2022).

The study was limited to an in vitro model using HMC‐3 cells. While in vitro models offer controlled experimental conditions and mechanistic insights, they may not fully represent the complex interactions and responses in vivo.

Our future research will focus on validating these findings in relevant in vivo models and exploring the translational potential of iron‐chelating agents and CIs. Additionally, we plan on investigating the impact of excess iron on other cell types within the CNS and its implications for overall brain homeostasis, which would further enhance our understanding of the broader consequences of iron dysregulation.

## 5 CONCLUSION

In conclusion, our study provides insight into the relationship between excess iron, cofilin activation, and microglial function in vitro. The therapeutic interventions of DFX and CI show promise in mitigating iron‐induced effects, offering potential avenues for developing targeted therapies for neuroinflammatory conditions associated with iron dysregulation. These findings contribute to the evolving landscape of neuroinflammation research, providing a foundation for future investigations aiming to unravel the complexities of iron and cofilin‐mediated microglial activation in neurological disorders.

## AUTHOR CONTRIBUTIONS

**Faheem Shehjar:** Conceptualization; methodology; investigation; validation; formal analysis; writing – original draft. **Antonisamy William James:** Methodology; data curation; visualization. **Reetika Mahajan:** Methodology; formal analysis; validation. **Zahoor A. Shah:** Conceptualization; methodology; validation; funding acquisition; resources; writing – review and editing; project administration.

## FUNDING INFORMATION

This research was funded by the National Institute of Neurological Disorders and Stroke of the National Institutes of Health #R01NS112642 to ZAS.

## CONFLICT OF INTEREST STATEMENT

The authors declare no conflicts of interest.

### PEER REVIEW

The peer review history for this article is available at https://www.webofscience.com/api/gateway/wos/peer‐review/10.1111/jnc.16260.

## Supporting information


Appendix S1.

## Data Availability

All data needed to assess the conclusions in the paper are exhibited in the paper.

## References

[jnc16260-bib-0001] Alaqel, S. I. , Dlamini, S. , Almarghalani, D. A. , Shettigar, A. , Alhadidi, Q. , Kodithuwakku, S. H. , Stary, C. , Tillekeratne, L. M. V. , & Shah, Z. A. (2022). Synthesis and development of a novel first‐in‐class cofilin inhibitor for neuroinflammation in hemorrhagic brain injury. ACS Chemical Neuroscience, 13, 1014–1029. 35302736 10.1021/acschemneuro.2c00010 PMC9996837

[jnc16260-bib-0002] Alhadidi, Q. , Bin Sayeed, M. S. , & Shah, Z. A. (2016). Cofilin as a promising therapeutic target for ischemic and hemorrhagic stroke. Translational Stroke Research, 7, 33–41. 26670926 10.1007/s12975-015-0438-2

[jnc16260-bib-0003] Alhadidi, Q. , & Shah, Z. A. (2018). Cofilin mediates LPS‐induced microglial cell activation and associated neurotoxicity through activation of NF‐κB and JAK‐STAT pathway. Molecular Neurobiology, 55, 1676–1691. 28194647 10.1007/s12035-017-0432-7 PMC5554748

[jnc16260-bib-0004] Aljuhani, M. , Ashraf, A. , Kislitsyna, E. , Anjum, R. , Hubens, C. , Parkes, H. , & So, P.‐W. (2020). Effects of iron and/or inflammation on regional brain magnetic resonance imaging T1 and T2 in an Alzheimer's disease mouse model. Alzheimer's & Dementia, 16, e040871.

[jnc16260-bib-0005] Alsegiani, A. S. , & Shah, Z. A. (2020). The role of cofilin in age‐related neuroinflammation. Neural Regeneration Research, 15, 1451–1459. 31997804 10.4103/1673-5374.274330 PMC7059588

[jnc16260-bib-0006] Alsegiani, A. S. M. (2022). Neuroinflammation and cognitive deficits in aging: Possible role of cofilin signaling. University of Toledo.

[jnc16260-bib-0007] Andersen, H. H. , Johnsen, K. B. , & Moos, T. (2014). Iron deposits in the chronically inflamed central nervous system and contributes to neurodegeneration. Cellular and Molecular Life Sciences, 71, 1607–1622. 24218010 10.1007/s00018-013-1509-8 PMC3983878

[jnc16260-bib-0008] Borst, K. , Dumas, A. A. , & Prinz, M. (2021). Microglia: Immune and non‐immune functions. Immunity, 54, 2194–2208. 34644556 10.1016/j.immuni.2021.09.014

[jnc16260-bib-0009] Chen, Y. , Fang, Z.‐M. , Yi, X. , Wei, X. , & Jiang, D.‐S. (2023). The interaction between ferroptosis and inflammatory signaling pathways. Cell Death & Disease, 14, 1–13. 36944609 10.1038/s41419-023-05716-0 PMC10030804

[jnc16260-bib-0010] Cowan, M. N. , Sethi, I. , & Harris, T. H. (2022). Microglia in CNS infections: Insights from *Toxoplasma gondii* and other pathogens. Trends in Parasitology, 38, 217–229. 35039238 10.1016/j.pt.2021.12.004 PMC8852251

[jnc16260-bib-0011] Dimauro, I. , Pearson, T. , Caporossi, D. , & Jackson, M. J. (2012). A simple protocol for the subcellular fractionation of skeletal muscle cells and tissue. BMC Research Notes, 5, 513. 22994964 10.1186/1756-0500-5-513 PMC3508861

[jnc16260-bib-0012] Donley, D. W. , Realing, M. , Gigley, J. P. , & Fox, J. H. (2021). Iron activates microglia and directly stimulates indoleamine‐2,3‐dioxygenase activity in the N171‐82Q mouse model of Huntington's disease. PLoS One, 16, e0250606. 33989290 10.1371/journal.pone.0250606 PMC8121302

[jnc16260-bib-0013] Gao, C. , Jiang, J. , Tan, Y. , & Chen, S. (2023). Microglia in neurodegenerative diseases: Mechanism and potential therapeutic targets. Signal Transduction and Targeted Therapy, 8, 1–37. 37735487 10.1038/s41392-023-01588-0 PMC10514343

[jnc16260-bib-0014] Jia, H. , Liu, X. , Cao, Y. , Niu, H. , Zhang, L. , Li, R. , Li, F. , Sun, D. , Shi, M. , Wa, L. , Liu, X. , Yang, G. , Chen, F. , Zhang, S. , & Zhang, J. (2023). Deferoxamine ameliorates neurological dysfunction by inhibiting ferroptosis and neuroinflammation after traumatic brain injury. Brain Research, 1812, 148383. 37149247 10.1016/j.brainres.2023.148383

[jnc16260-bib-0015] Kenkhuis, B. , Somarakis, A. , de Haan, L. , Dzyubachyk, O. , IJsselsteijn, M. E. , de Miranda, N. F. C. C. , Lelieveldt, B. P. F. , Dijkstra, J. , van Roon‐Mom, W. M. C. , Höllt, T. , & van der Weerd, L. (2021). Iron loading is a prominent feature of activated microglia in Alzheimer's disease patients. Acta Neuropathologica Communications, 9, 27. 33597025 10.1186/s40478-021-01126-5 PMC7887813

[jnc16260-bib-0016] Kenkhuis, B. , van Eekeren, M. , Parfitt, D. A. , Ariyurek, Y. , Banerjee, P. , Priller, J. , van der Weerd, L. , & van Roon‐Mom, W. M. C. (2022). Iron accumulation induces oxidative stress, while depressing inflammatory polarization in human iPSC‐derived microglia. Stem Cell Reports, 17, 1351–1365. 35523178 10.1016/j.stemcr.2022.04.006 PMC9213827

[jnc16260-bib-0017] Lapeña‐Luzón, T. , Rodríguez, L. R. , Beltran‐Beltran, V. , Benetó, N. , Pallardó, F. V. , & Gonzalez‐Cabo, P. (2021). Cofilin and neurodegeneration: New functions for an old but gold protein. Brain Sciences, 11, 954. 34356188 10.3390/brainsci11070954 PMC8303701

[jnc16260-bib-0018] Levi, S. , Ripamonti, M. , Moro, A. S. , & Cozzi, A. (2024). Iron imbalance in neurodegeneration. Molecular Psychiatry, 29, 1–14. 38212377 10.1038/s41380-023-02399-z PMC11176077

[jnc16260-bib-0019] Li, Z. , Liu, Y. , Wei, R. , Khan, S. , Zhang, R. , Zhang, Y. , Yong, V. W. , & Xue, M. (2022). Iron neurotoxicity and protection by deferoxamine in intracerebral hemorrhage. Frontiers in Molecular Neuroscience, 15, 927334. 35782383 10.3389/fnmol.2022.927334 PMC9245523

[jnc16260-bib-0020] Liu, S. , Gao, X. , & Zhou, S. (2022). New target for prevention and treatment of neuroinflammation: Microglia iron accumulation and ferroptosis. ASN Neuro, 14, 17590914221133236. 36285433 10.1177/17590914221133236 PMC9607999

[jnc16260-bib-0021] Long, H.‐Z. , Zhou, Z.‐W. , Cheng, Y. , Luo, H.‐Y. , Li, F.‐J. , Xu, S.‐G. , & Gao, L.‐C. (2022). The role of microglia in Alzheimer's disease from the perspective of immune inflammation and iron metabolism. Frontiers in Aging Neuroscience, 14, 888989. 35847685 10.3389/fnagi.2022.888989 PMC9284275

[jnc16260-bib-0022] Madineni, A. , Alhadidi, Q. , & Shah, Z. A. (2016). Cofilin inhibition restores neuronal cell death in oxygen‐glucose deprivation model of ischemia. Molecular Neurobiology, 53, 867–878. 25526862 10.1007/s12035-014-9056-3 PMC4475502

[jnc16260-bib-0023] Marchenko, S. , & Flanagan, L. (2007). Immunocytochemistry: Human neural stem cells. Journal of Visualized Experiments, 7, 267. 10.3791/267 PMC2565851 18989438

[jnc16260-bib-0024] Messa, E. , Defilippi, I. , Roetto, A. , Messa, F. , Arruga, F. , Carturan, S. , Rosso, V. , Bracco, E. , Cilloni, D. , & Saglio, G. (2008). Deferasirox is the only iron chelator acting as a potent NF‐KB inhibitor in myelodysplastic syndromes. Blood, 112, 2671.

[jnc16260-bib-0025] Minamide, L. S. , Striegl, A. M. , Boyle, J. A. , Meberg, P. J. , & Bamburg, J. R. (2000). Neurodegenerative stimuli induce persistent ADF/cofilin–actin rods that disrupt distal neurite function. Nature Cell Biology, 2, 628–636. 10980704 10.1038/35023579

[jnc16260-bib-0026] Ndayisaba, A. , Kaindlstorfer, C. , & Wenning, G. K. (2019). Iron in neurodegeneration—Cause or consequence? Frontiers in Neuroscience, 13, 180. 30881284 10.3389/fnins.2019.00180 PMC6405645

[jnc16260-bib-0027] Prinz, M. , Jung, S. , & Priller, J. (2019). Microglia biology: One century of evolving concepts. Cell, 179, 292–311. 31585077 10.1016/j.cell.2019.08.053

[jnc16260-bib-0028] Rathnasamy, G. , Ling, E.‐A. , & Kaur, C. (2011). Iron and iron regulatory proteins in amoeboid microglial cells are linked to oligodendrocyte death in hypoxic neonatal rat periventricular white matter through production of proinflammatory cytokines and reactive oxygen/nitrogen species. The Journal of Neuroscience, 31, 17982–17995. 22159112 10.1523/JNEUROSCI.2250-11.2011 PMC6634148

[jnc16260-bib-0029] Riederer, P. , Nagatsu, T. , Youdim, M. B. H. , Wulf, M. , Dijkstra, J. M. , & Sian‐Huelsmann, J. (2023). Lewy bodies, iron, inflammation and neuromelanin: Pathological aspects underlying Parkinson's disease. Journal of Neural Transmission, 130, 627–646. 37062012 10.1007/s00702-023-02630-9 PMC10121516

[jnc16260-bib-0030] Ryan, S. K. , Zelic, M. , Han, Y. , Teeple, E. , Chen, L. , Sadeghi, M. , Shankara, S. , Guo, L. , Li, C. , Pontarelli, F. , Jensen, E. H. , Comer, A. L. , Kumar, D. , Zhang, M. , Gans, J. , Zhang, B. , Proto, J. D. , Saleh, J. , Dodge, J. C. , … Hammond, T. R. (2023). Microglia ferroptosis is regulated by SEC24B and contributes to neurodegeneration. Nature Neuroscience, 26, 12–26. 36536241 10.1038/s41593-022-01221-3 PMC9829540

[jnc16260-bib-0031] Samstag, Y. , John, I. , & Wabnitz, G. H. (2013). Cofilin: A redox sensitive mediator of actin dynamics during T‐cell activation and migration. Immunological Reviews, 256, 30–47. 24117811 10.1111/imr.12115 PMC3884758

[jnc16260-bib-0032] Shao, F. , Wang, X. , Wu, H. , Wu, Q. , & Zhang, J. (2022). Microglia and neuroinflammation: Crucial pathological mechanisms in traumatic brain injury‐induced neurodegeneration. Frontiers in Aging Neuroscience, 14, 825086. 35401152 10.3389/fnagi.2022.825086 PMC8990307

[jnc16260-bib-0033] Shehjar, F. , Almarghalani, D. A. , Mahajan, R. , Hasan, S. A.‐M. , & Shah, Z. A. (2024). The multifaceted role of cofilin in neurodegeneration and stroke: Insights into pathogenesis and targeting as a therapy. Cells, 13, 188. 38247879 10.3390/cells13020188 PMC10814918

[jnc16260-bib-0034] Thomsen, M. S. , Andersen, M. V. , Christoffersen, P. R. , Jensen, M. D. , Lichota, J. , & Moos, T. (2015). Neurodegeneration with inflammation is accompanied by accumulation of iron and ferritin in microglia and neurons. Neurobiology of Disease, 81, 108–118. 25801802 10.1016/j.nbd.2015.03.013

[jnc16260-bib-0035] van Rheenen, J. , Condeelis, J. , & Glogauer, M. (2009). A common cofilin activity cycle in invasive tumor cells and inflammatory cells. Journal of Cell Science, 122, 305–311. 19158339 10.1242/jcs.031146 PMC2772875

[jnc16260-bib-0036] Vela, D. (2018). The dual role of hepcidin in brain iron load and inflammation. Frontiers in Neuroscience, 12, 740. 30374287 10.3389/fnins.2018.00740 PMC6196657

[jnc16260-bib-0037] Wang, Q. , Yuan, W. , Yang, X. , Wang, Y. , Li, Y. , & Qiao, H. (2020). Role of cofilin in Alzheimer's disease. Frontiers in Cell and Development Biology, 8, 584898. 10.3389/fcell.2020.584898 PMC7726191 33324642

[jnc16260-bib-0038] Ward, R. J. , Dexter, D. T. , & Crichton, R. R. (2022). Iron, neuroinflammation and neurodegeneration. International Journal of Molecular Sciences, 23, 7267. 35806270 10.3390/ijms23137267 PMC9266893

[jnc16260-bib-0039] Yan, M. , Xiong, M. , Dai, L. , Zhang, X. , Zha, Y. , Deng, X. , Yu, Z. , & Zhang, Z. (2022). Cofilin 1 promotes the pathogenicity and transmission of pathological α‐synuclein in mouse models of Parkinson's disease. npj Parkinson's Disease, 8, 1–11. 10.1038/s41531-021-00272-w PMC8748615 35013321

[jnc16260-bib-0040] Yu, H. , Lin, L. , Zhang, Z. , Zhang, H. , & Hu, H. (2020). Targeting NF‐κB pathway for the therapy of diseases: Mechanism and clinical study. Signal Transduction and Targeted Therapy, 5, 1–23. 32958760 10.1038/s41392-020-00312-6 PMC7506548

