# Supplementary material for: Inhibition of iron‐induced cofilin activation and inflammation in microglia by a novel cofilin inhibitor
Source: J Neurochem. 2024 Nov 18;169(2):e16260. doi: 10.1111/jnc.16260 (PMC11808637; doi:10.1111/jnc.16260)
Supplement: Supplementary file 1 — Appendix S1. [file JNC-169-0-s001.zip › 3_Supplementary JNC revised FS 11-12-24-ZAS.pdf]

Inhibition of Iron-Induced Cofilin Activation and Inflammation in Microglial Cells by a Novel Cofilin Inhibitor

Faheem Shehjar, Antonisamy William James, Reetika Mahajan, Zahoor A Shah¶

Department of Medicinal and Biological Chemistry, College of Pharmacy and Pharmaceutical Sciences, Toledo, Ohio 43614

Supplementary Material Figure 1

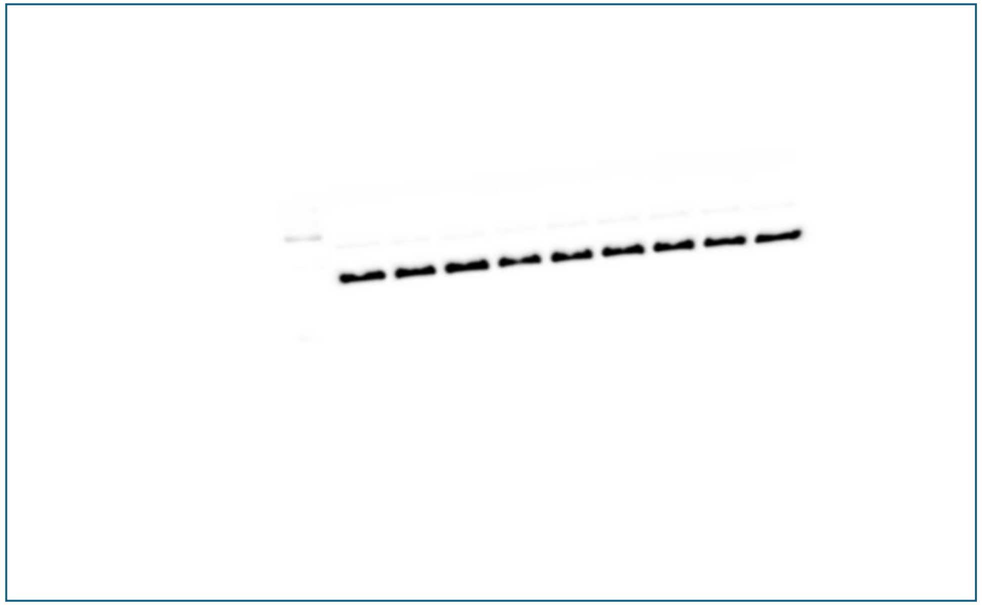

Figure 1 B (β-actin)

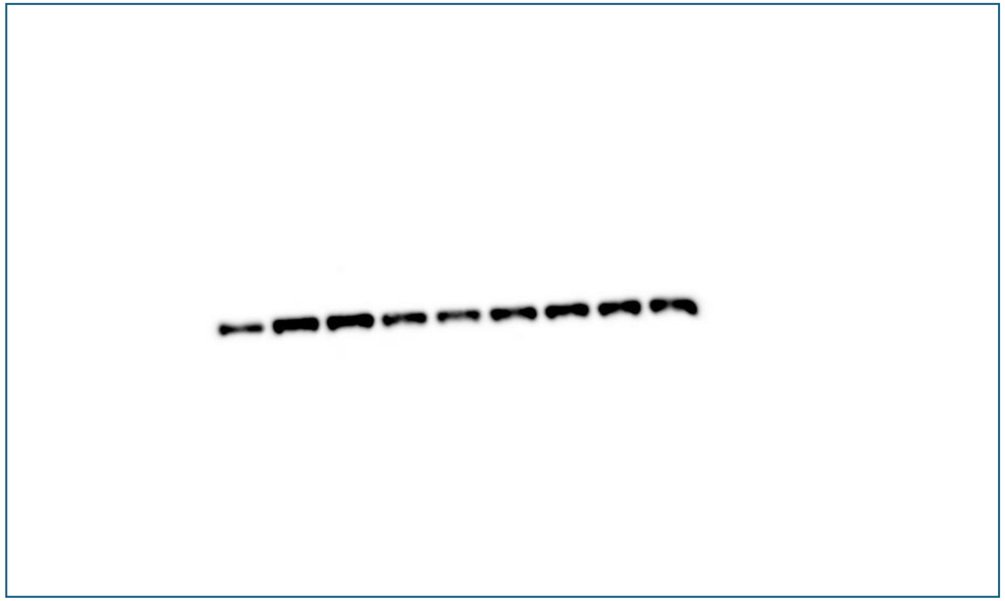

Figure 1 B (Cofilin)

Blots showing expression of β-actin (loading control) and cofilin with different concentrations of $FeSO_4$ & DFX.

**Bands**	**1**	**2**	**3**	**4**	**5**	**6**	**7**	**8**	**9**
**FeSo4 (μM)**	**C**	**100**	**200**	**300**	**400**	**100**	**200**	**300**	**400**
**DFX (150μM)**	**-**	**-**	**-**	**-**	**-**	**+**	**+**	**+**	**+**

Supplementary Material Figure 2A

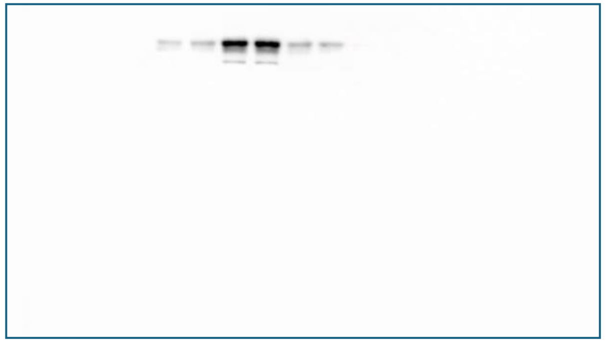

FTH 48 hours

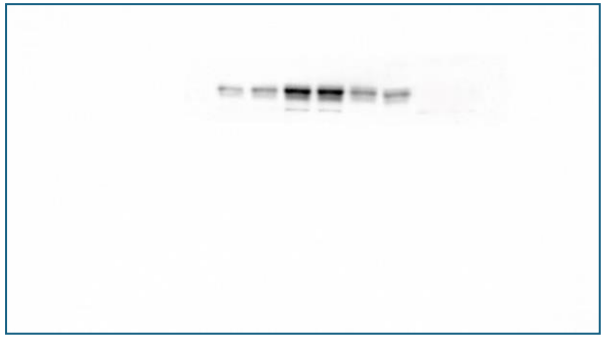

FTH 24hours

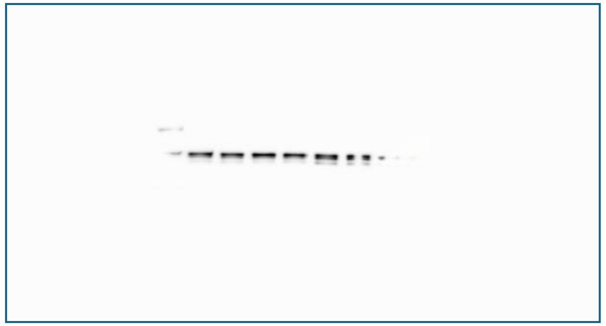

β-actin 72 hours

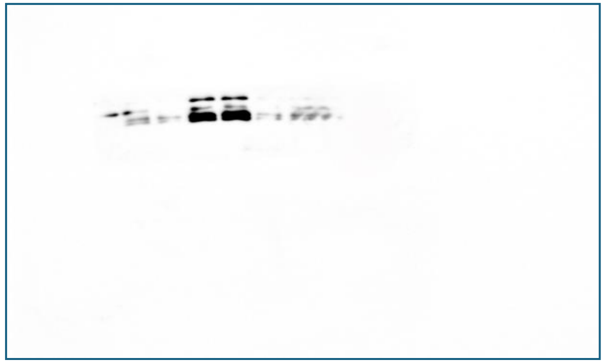

FTH 72 hours

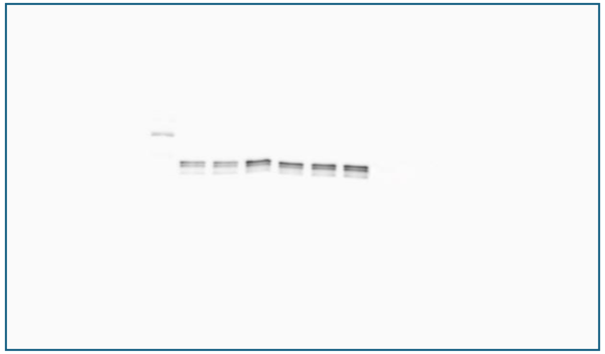

β-actin 24 hours

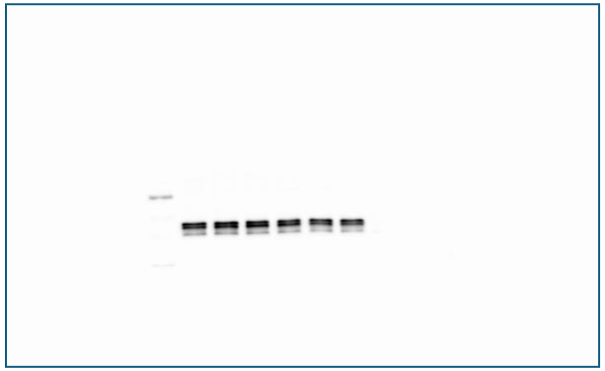

β-actin 48 hours

Blots showing ferritin heavy chain & β-actin (loading control) expression in HMC-3 cells treated with $FeSO_4$ (100µM) and DFX (150µM) at different time points. First two bands represent control, 3rd & 4th band represent $FeSO_4$ treatment, 5th & 6th bands represent $FeSO_4$+DFX co-treatment.

Supplementary Material Figure 2B

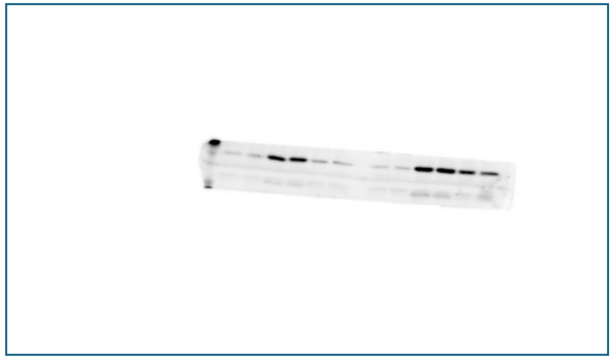

FTL 48 hours (right lanes)

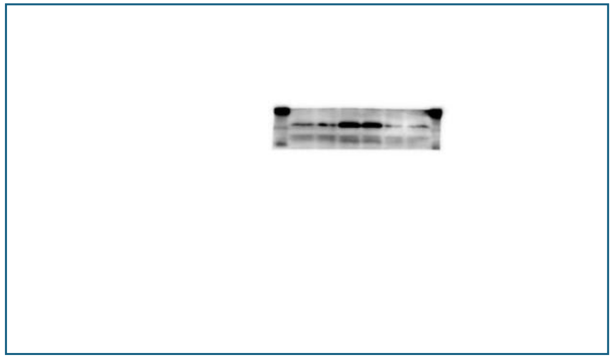

FTL 24hours

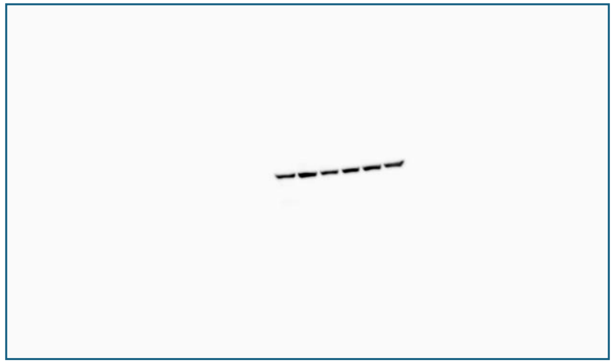

β-actin 72 hours

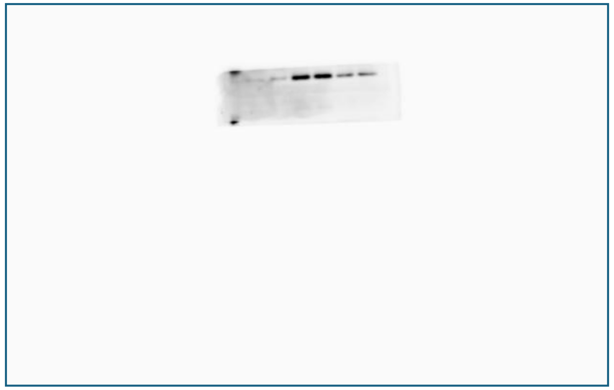

FTL 72 hours

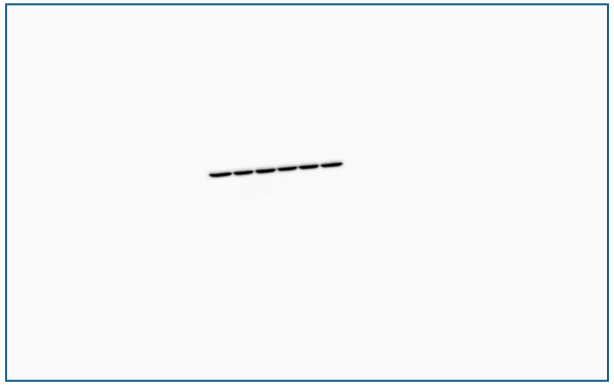

β-actin 24 hours

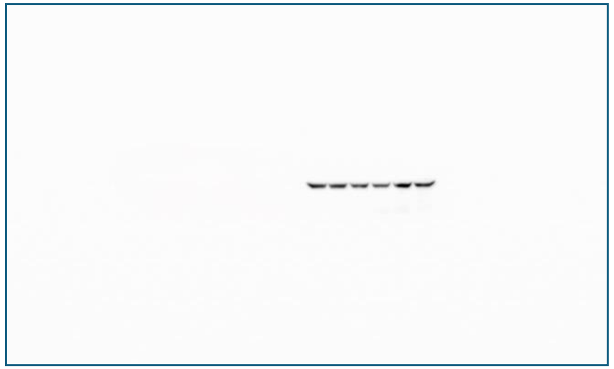

β-actin 48 hours

Blots showing ferritin light chain & β-actin (loading control) expression in HMC-3 cells treated with FeSO4 (100μM) and DFX (150μM) at different time points. First two bands represent control, 3rd & 4th band represent FeSO4 treatment, 5th & 6th bands represent FeSO4+DFX co-treatment.

Supplementary Material Figure 3A

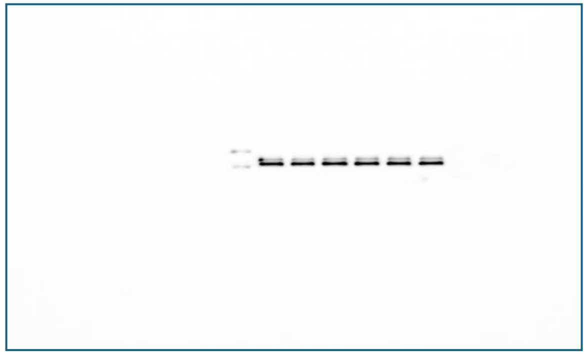

β-actin 24 hours

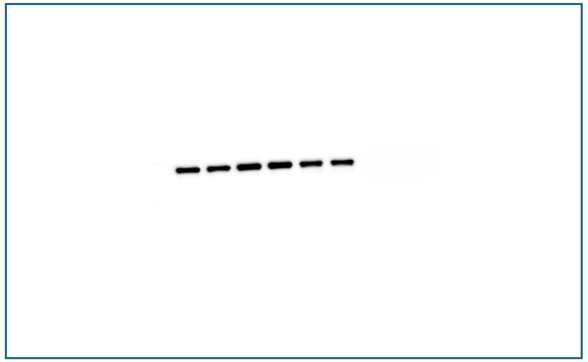

β-actin 48 hours

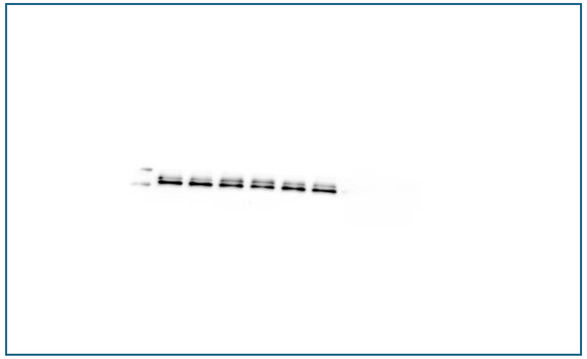

β-actin 72 hours

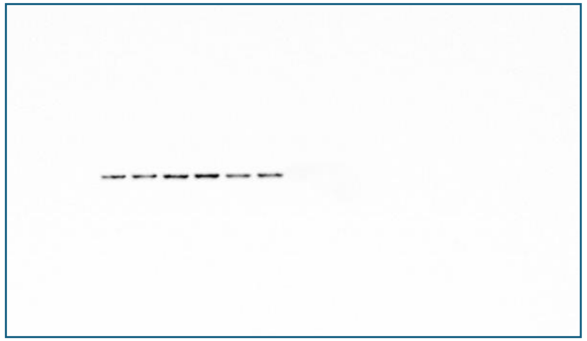

Cofilin 24 hours

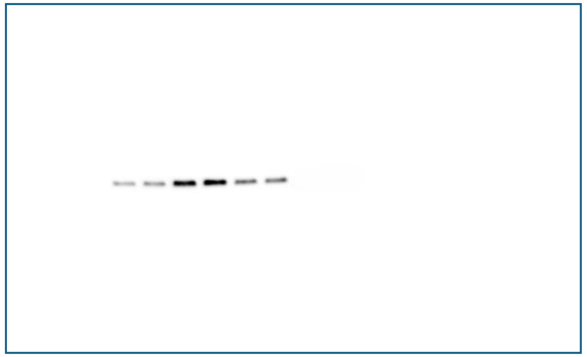

Cofilin 48 hours

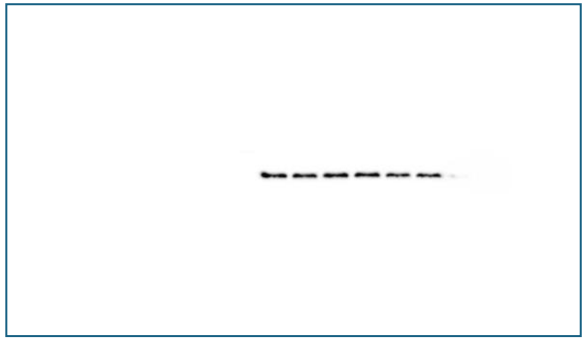

Cofilin 72 hours

Blots showing cofilin & β-actin (loading control) expression in HMC-3 cells treated with $FeSO_4$ (100µM) and DFX (150µM) at different time points. First two bands represent control, 3rd & 4th band represent $FeSO_4$ treatment, 5th & 6th bands represent $FeSO_4$+DFX co-treatment.

Supplementary Material Figure 3B

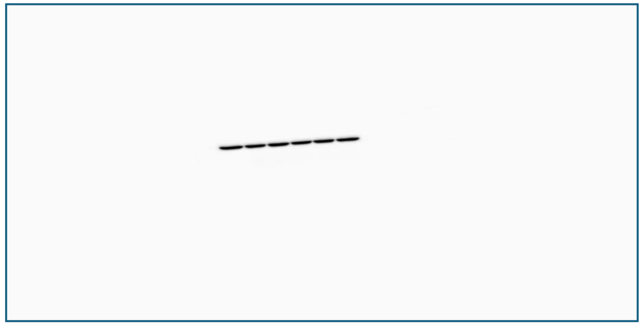

β-actin 24 hours

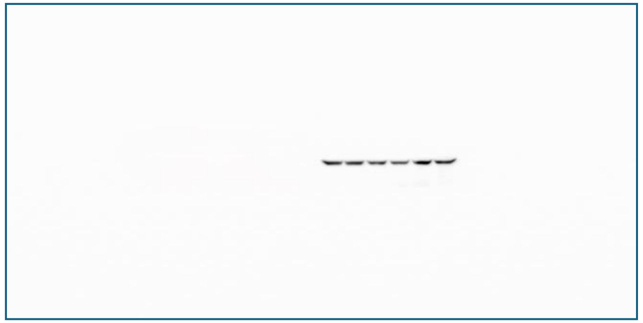

β-actin 48 hours

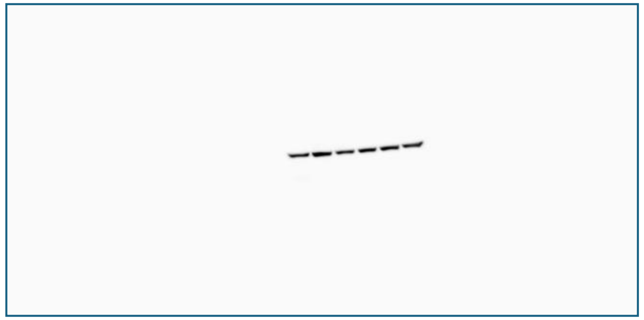

β-actin 72 hours

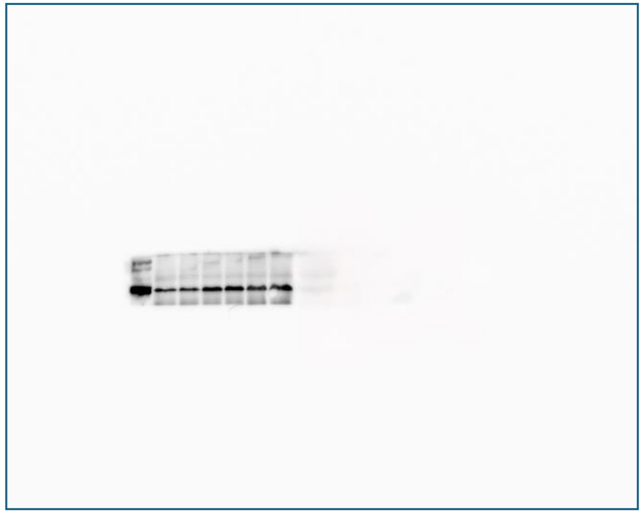

DMT-1 24 hours

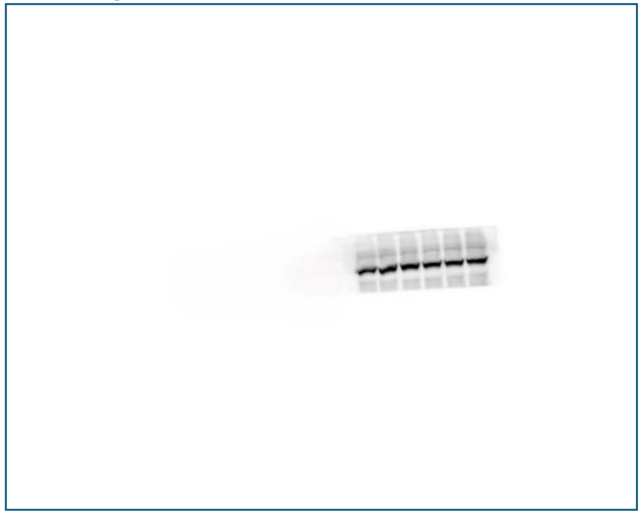

DMT-1 48 hours

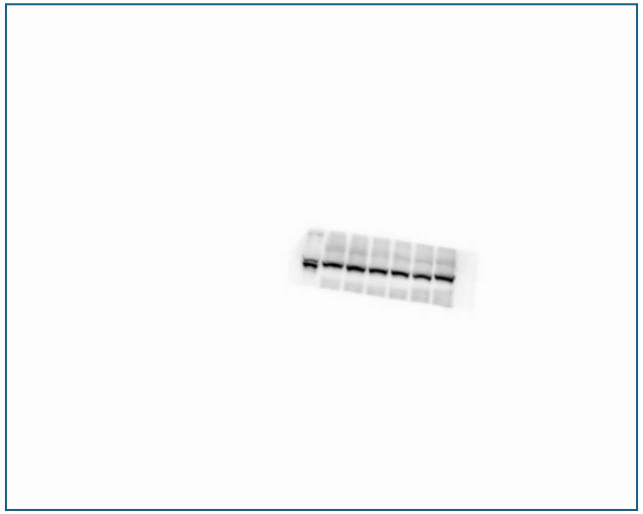

DMT-1 72 hours

Blots showing DMT-1 & β-actin (loading control) expression in HMC-3 cells treated with FeSO4 (100μM) and DFX (150μM) at different time points. First two bands represent control, 3rd & 4th band represent FeSO4 treatment, 5th & 6th bands represent FeSO4+DFX co- treatment.

Supplementary Material Figure 4A

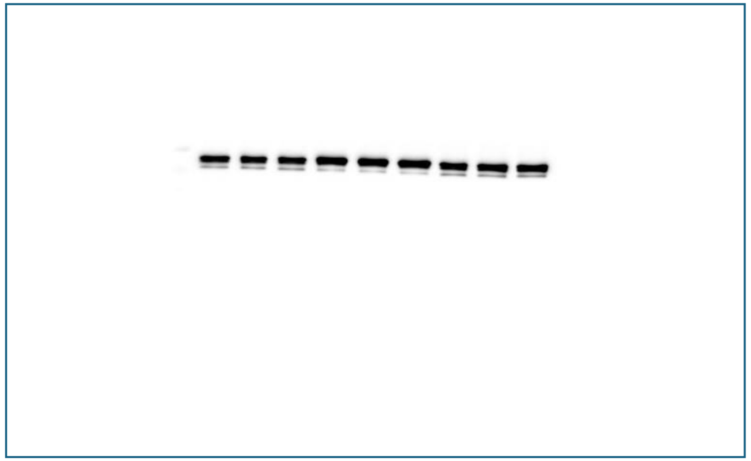

β-actin

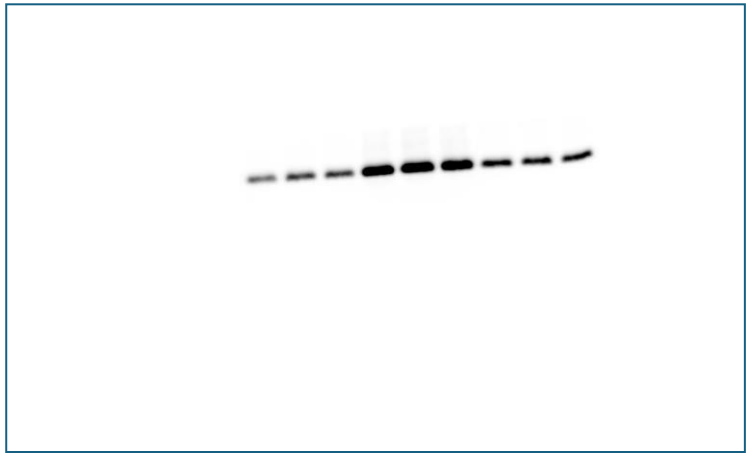

pCofilin

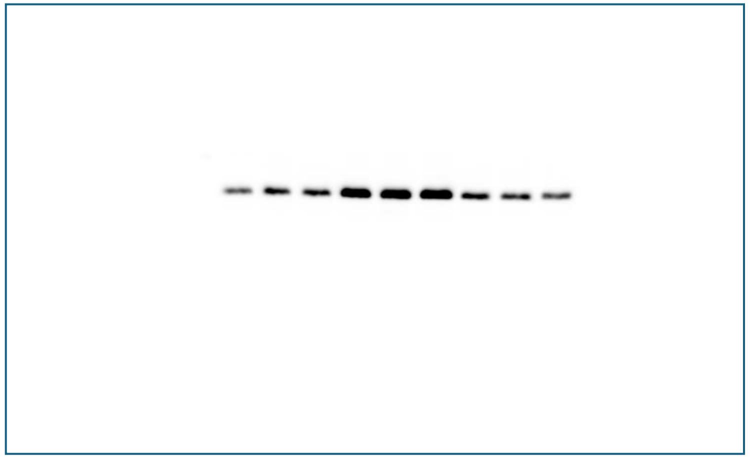

Cofilin

Blots showing β-actin (loading control), p-cofilin and cofilin expression in response to FeSO4 exposure and DFX treatment. First three bands represent control, bands 4, 5 & 6 represent FeSO4 (100uM) treatment, bands 7, 8 & 9 represent FeSO4+DFX treatment.

Supplementary Material Figure 4B

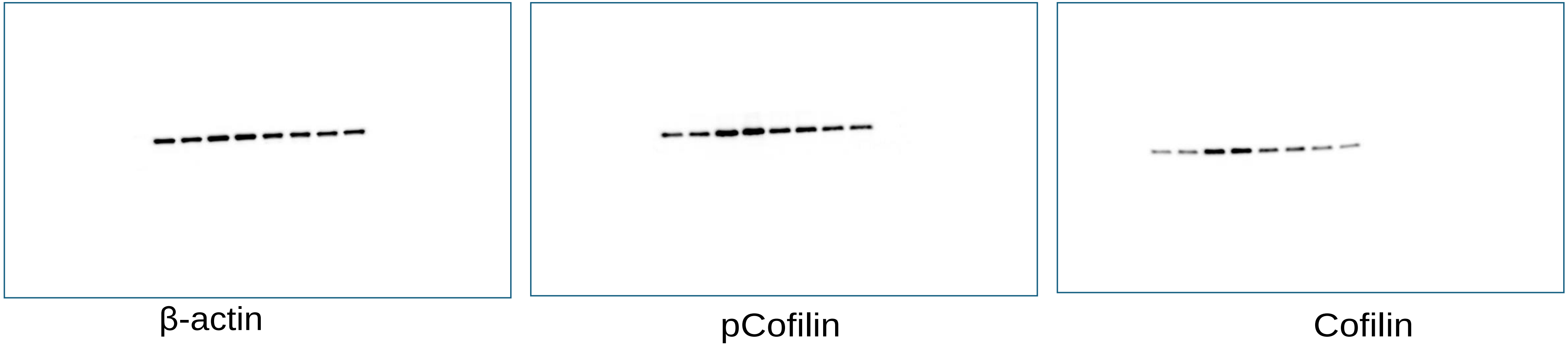

Blots showing β-actin (loading control), p-cofilin and cofilin expression in response to $FeSO_4$ exposure and DFX/CI treatment. First two bands represent control, bands 3 & 4 represent $FeSO_4$ (100µM) treatment, bands 5 & 6 represent $FeSO_4$+DFX treatment, bands 7 & 8 represent $FeSO_4$+CI treatment .

Supplementary Material Figure 5

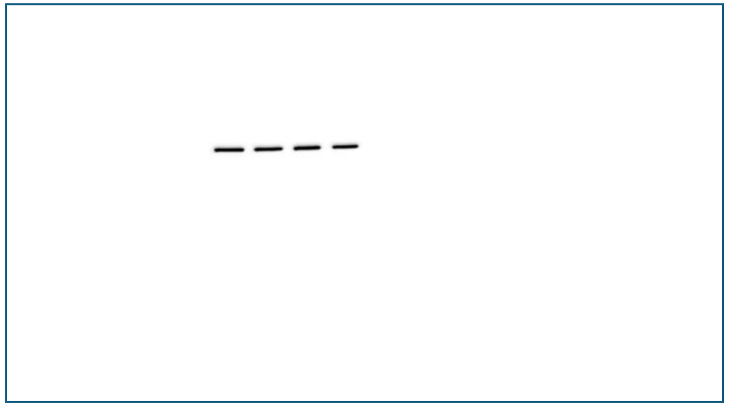

β-actin cytosol

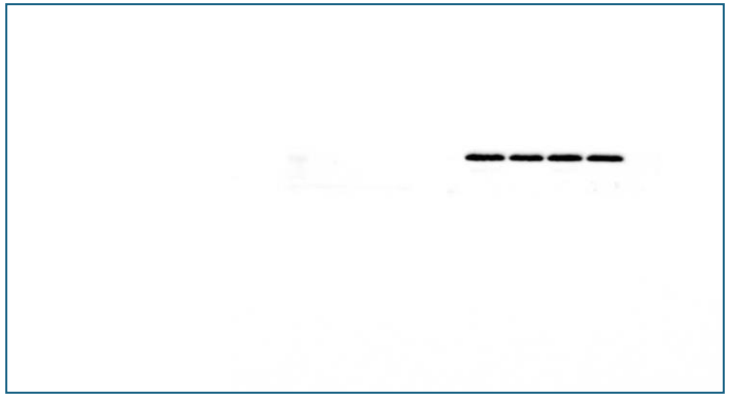

Histone Nuclear

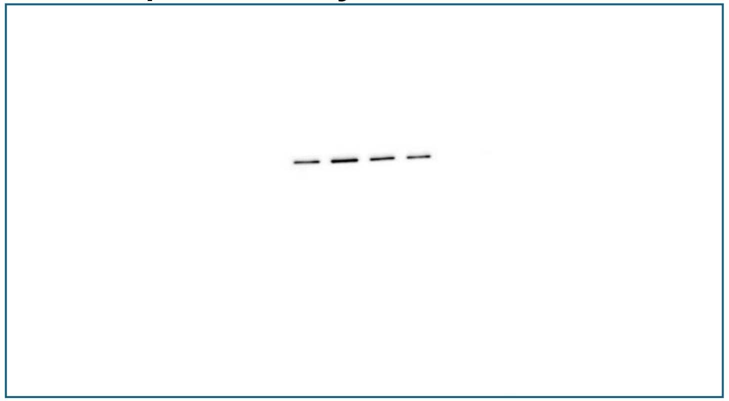

NFkB cytosol

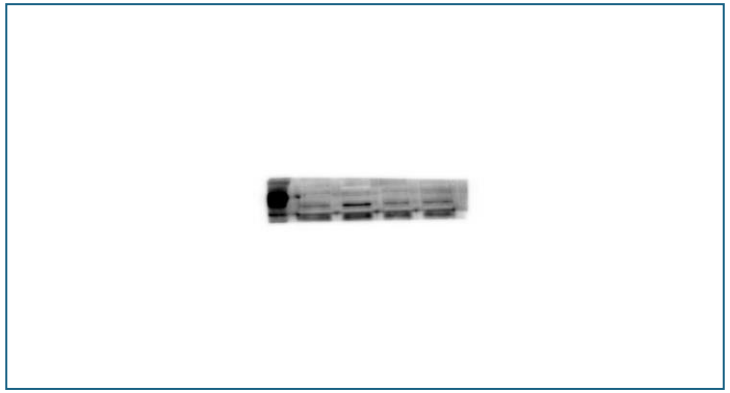

NFkB Nuclear

For figure 8 of main manuscript. Blots showing over-expression of NF-kB in cytosolic and nuclear fractions of HMC-3 cells in response to FeSO4 exposure and treatment with DFX and CI (β-actin & Histone served as loading controls for cytosolic & nuclear fractions respectively). Band 1 represents control, band 2 represents FeSO4 treatment, band 3 represents DFX cotreatment, band 4 represents CI co-

Certificate of Analysis HMC-3 ATCC

ATCC®

CERTIFICATE OF ANALYSIS

**ATCC® Number:**	**CRL-3304™**
**Lot Number:**	**70043235**
**Name:**	**HMC3**
**Description:**	**Embryonic Microglia Clone 3**
**Species:**	**Human *(Homo sapiens)***
**Volume/Ampule:**	**Approximately 1 mL**
**Date Frozen:**	**05APR2021**
**Recovery:**	**A T-75 setup at a seeding density of 2.0 x 10^4 viable cells/cm^2 reaches approximately 40% confluence in 1 day and 90% confluence in 3 days.**
**Product Format:**	**Cells cryopreserved in the appropriate cryopreservation medium**
**Expiration Date:**	**Not applicable**
**Storage Conditions:**	**Vapor phase of liquid nitrogen**

Test / Method	Specification	Result
**Ampule passage number**	Report results	Unknown + 7
**Population doubling level (PDL)**	Report results	Not applicable
**Total cells/ampule**<br>(Cell count using Trypan Blue stain method)	Report results	1.9 x 10^6 total cells/ampule
**Post-freeze viability**<br>(Cell count using Trypan Blue stain method)	≥ 50.0%	92.1%
**Growth properties**<br>(Visual observation method)	Adherent	Adherent
**Morphology**<br>(Visual observation method)	Epithelial-Like*	Epithelial-Like
**Test for mycoplasma contamination**<br>Hoechst DNA stain (indirect) method<br>Agar culture (direct) method<br>PCR-based assay	 None detected<br>None detected<br>None detected	 None detected<br>None detected<br>None detected
**Species determination: COI assay (interspecies)**	Human	Human

ATCC
10801 University Boulevard
Manassas, VA 20110-2209 USA
www.atcc.org

800-638-6597 or 703-365-2700
Fax: 703-365-2750
E-mail: tech@atcc.org
or contact your local distributor
