# Supplementary material for: Inhibition of iron‐induced cofilin activation and inflammation in microglia by a novel cofilin inhibitor
Source: J Neurochem. 2024 Nov 18;169(2):e16260. doi: 10.1111/jnc.16260 (PMC11808637; doi:10.1111/jnc.16260)
Supplement: Supplementary file 1 — Appendix S1. [file JNC-169-0-s001.zip › SUPPLEMENTARY JNC.pdf]

Inhibition of Iron-Induced Cofilin Activation and Inflammation in Microglial Cells by a Novel Cofilin Inhibitor

Faheem Shehjar, Antonisamy William James, Reetika Mahajan, Zahoor A Shah[¶]

Department of Medicinal and Biological Chemistry, College of Pharmacy and Pharmaceutical Sciences, Toledo, Ohio 43614

Supplementary material Figure 1

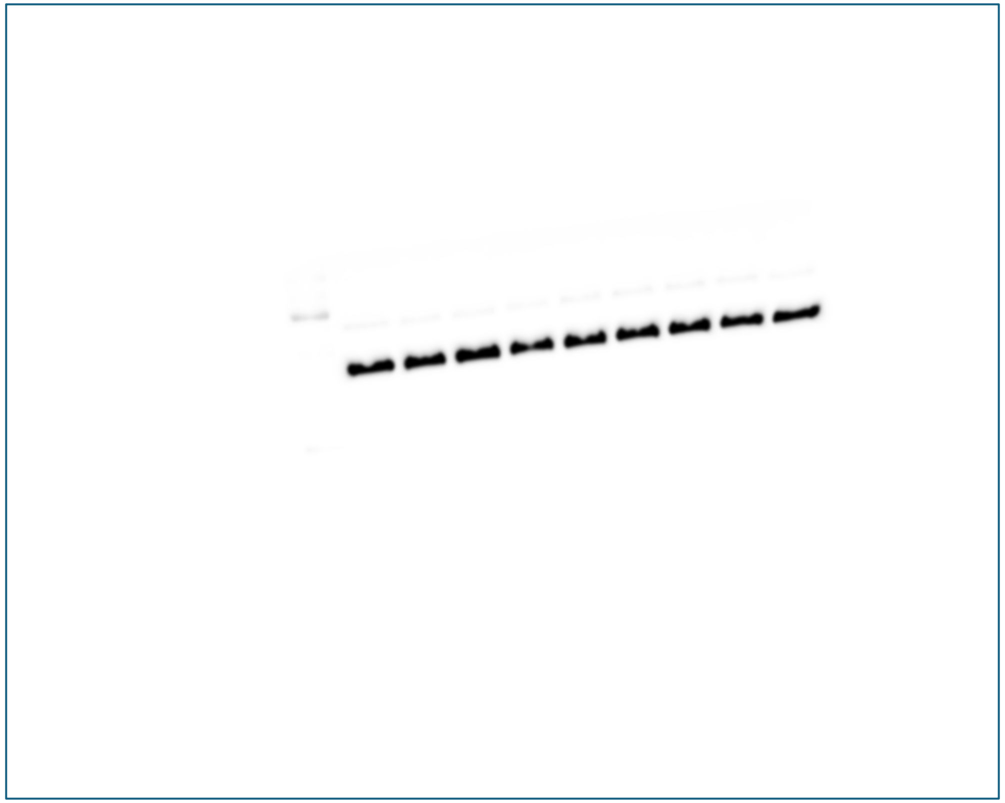

Figure 1 B (Beta actin

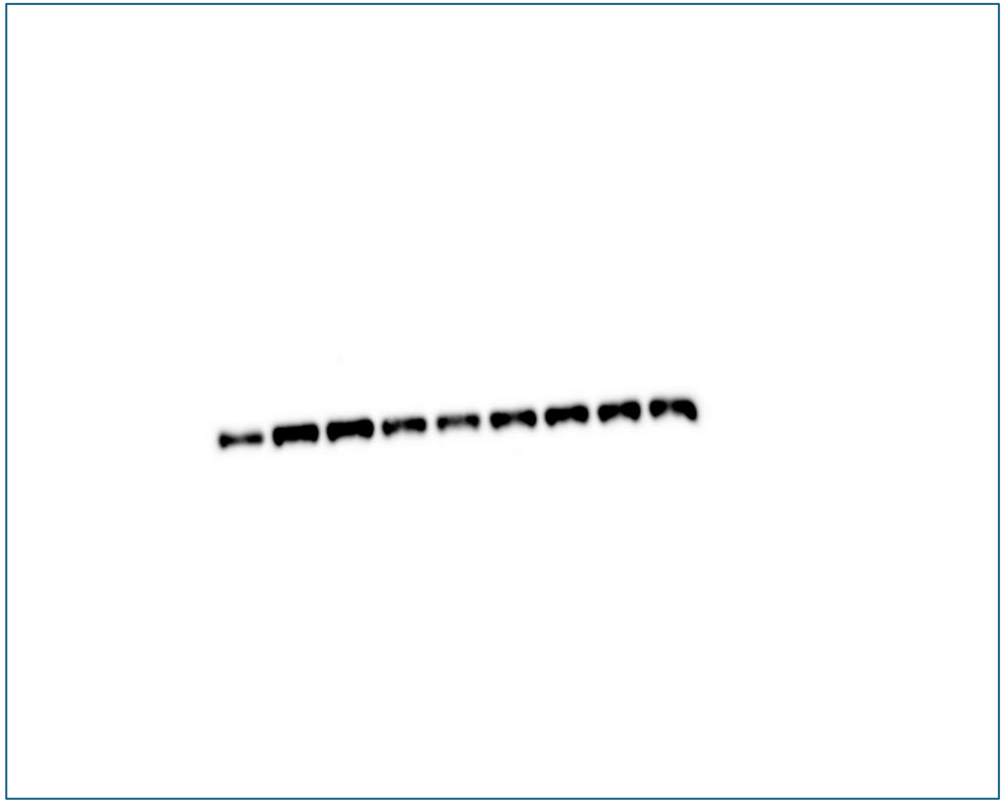

Figure 1 B (Cofilin)

Supplementary material Figure 2 A

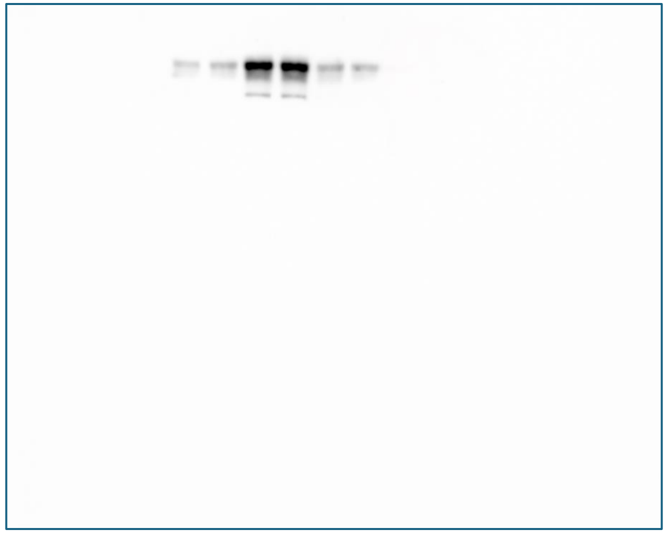

FTH 48 hours

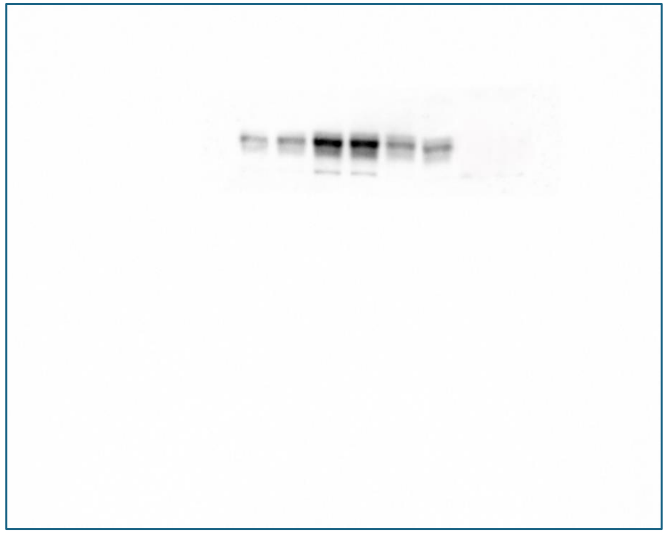

FTH 24hours

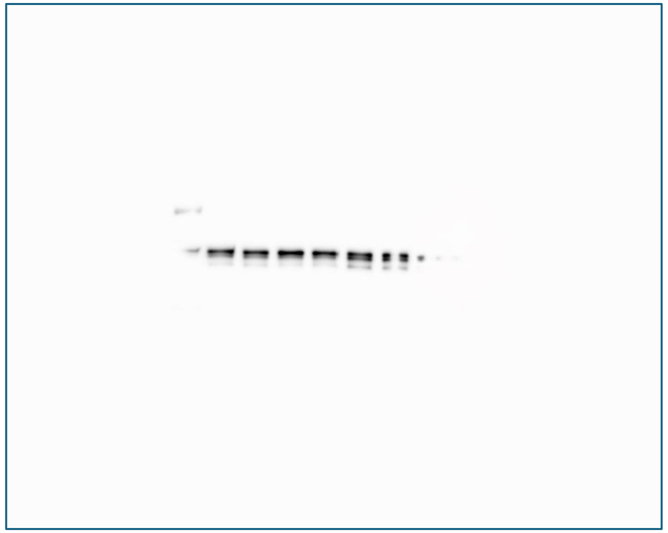

Beta actin 72 hours

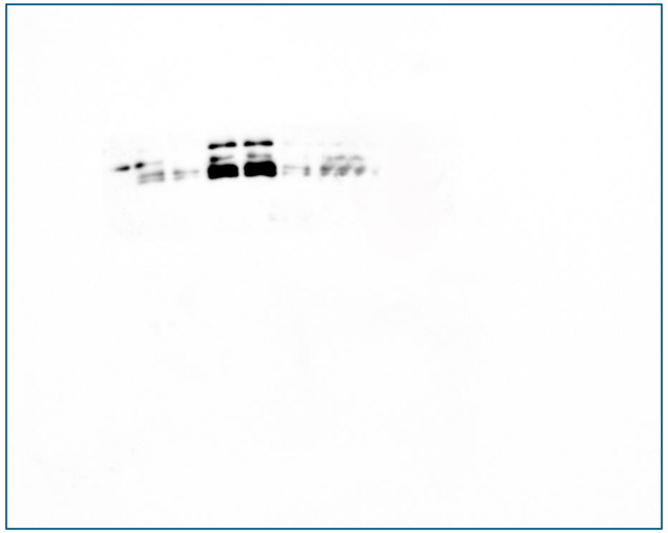

FTH 72 hours

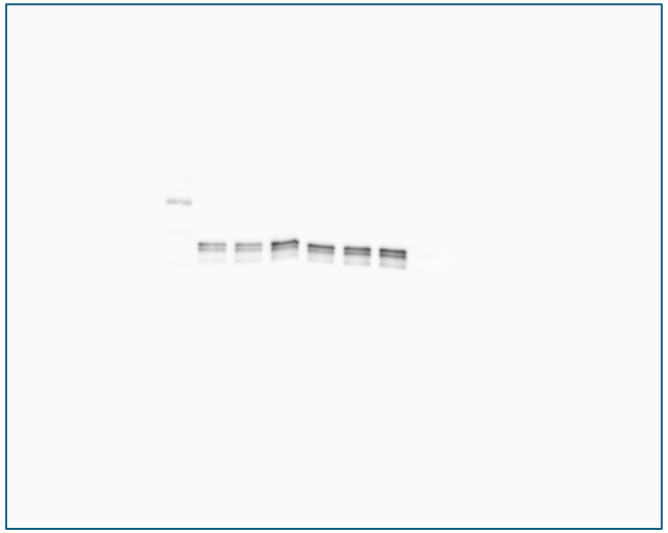

Beta actin 24 hours

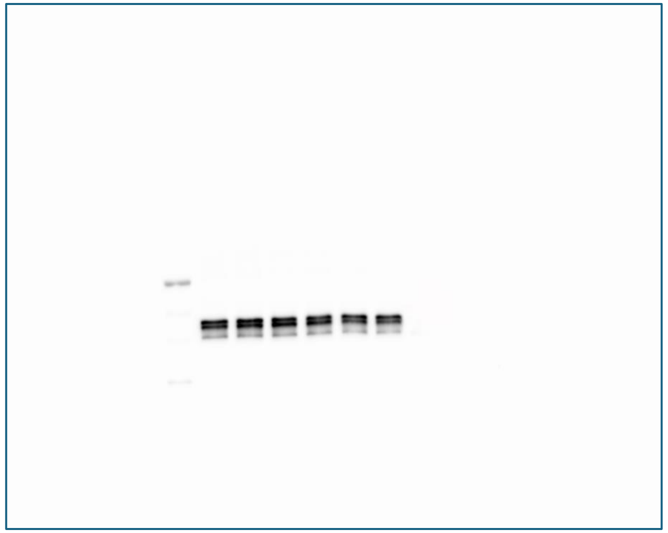

Beta actin 48 hours

Supplementary material Figure 2 B

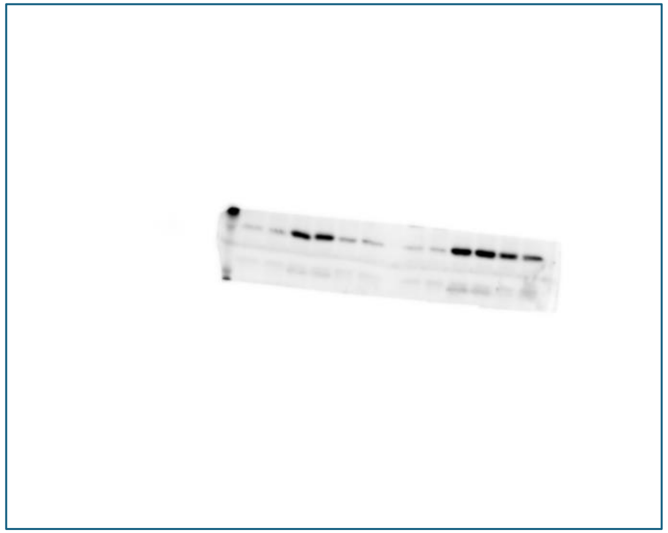

FTL 48 hours (right lanes)

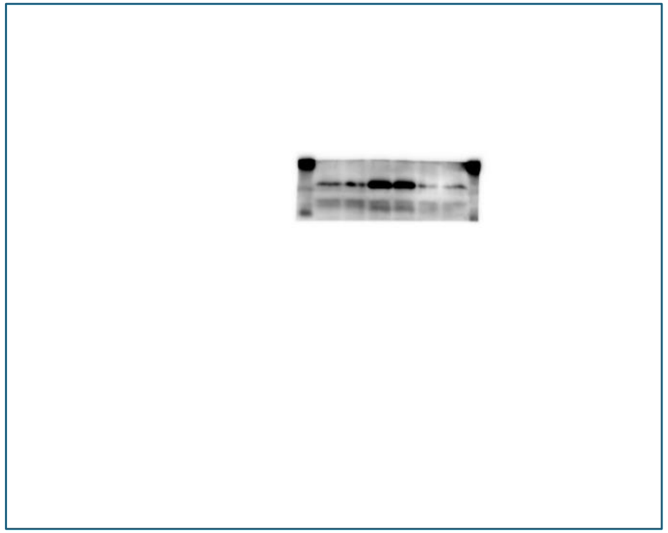

FTL 24hours

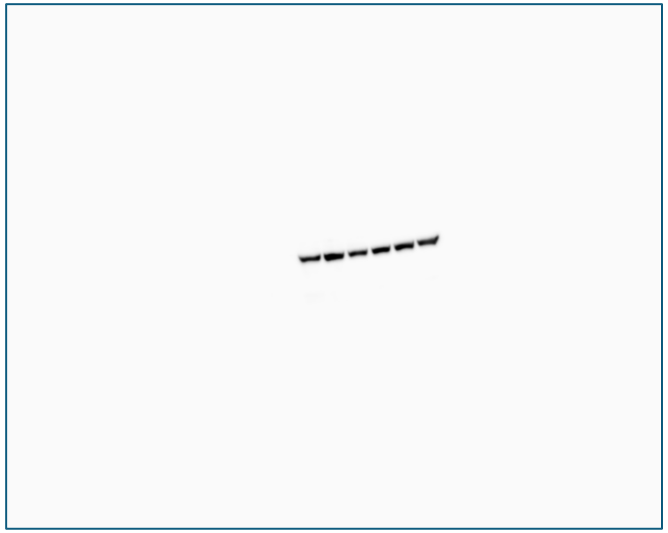

Beta actin 72 hours

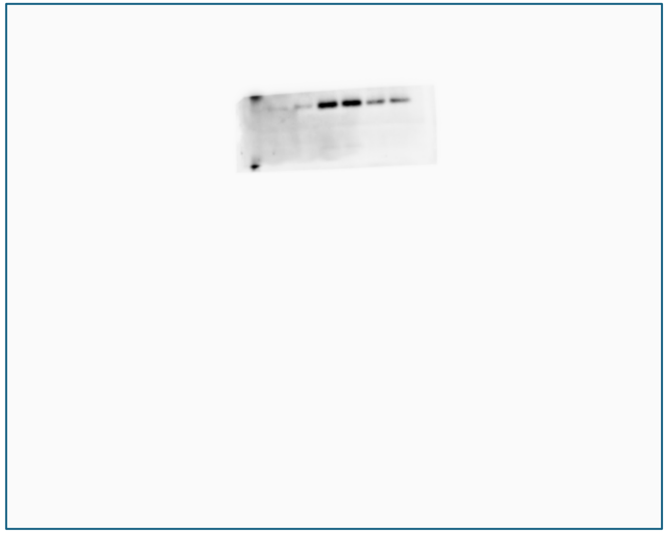

FTL 72 hours

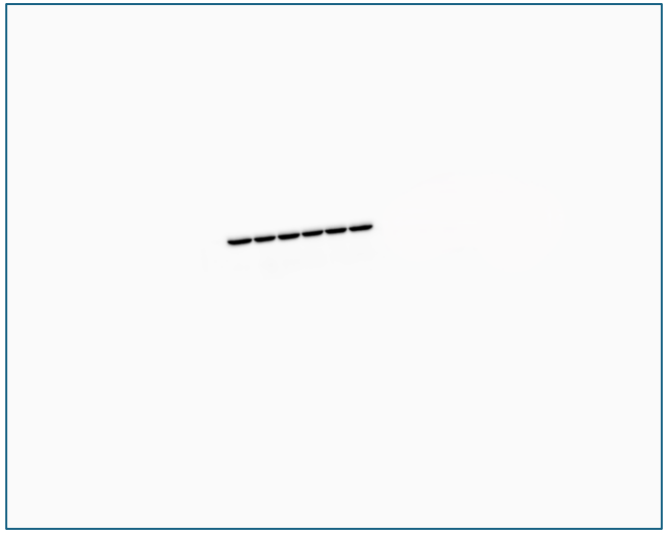

Beta actin 24 hours

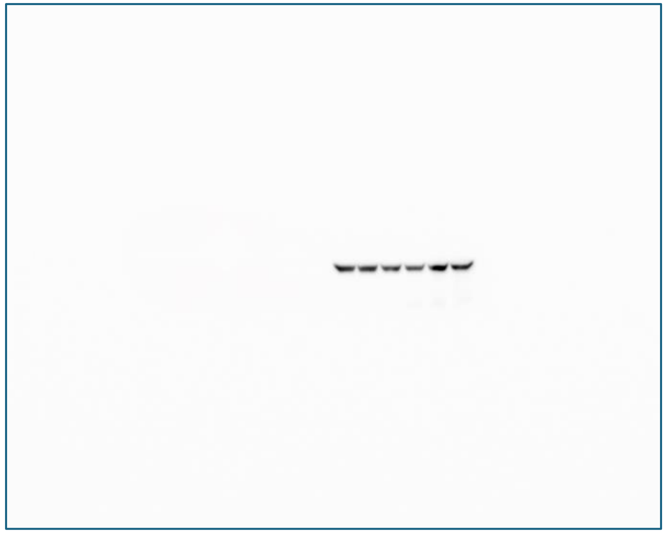

Beta actin 48 hours

Supplementary material Figure 3 A

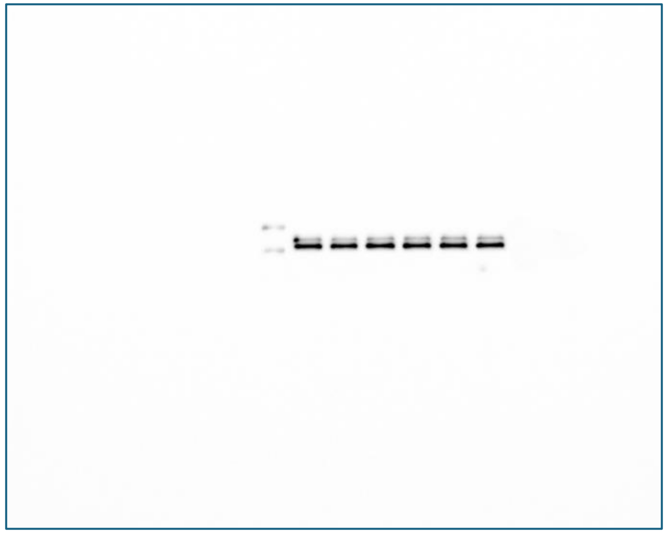

Beta actin 24 hours

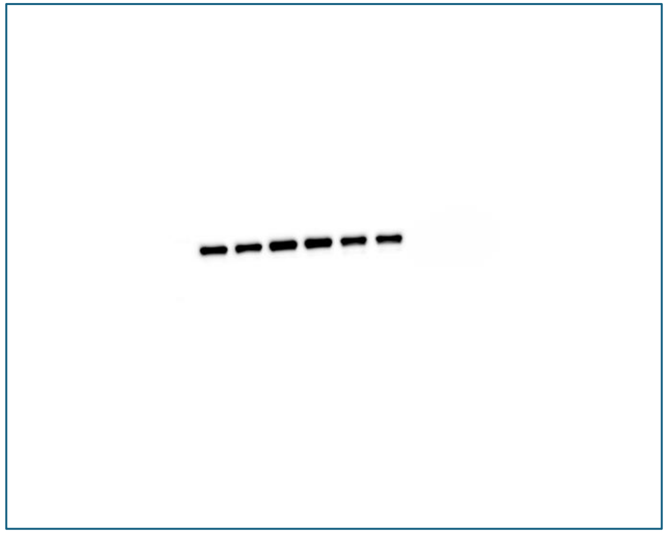

Beta actin 48 hours

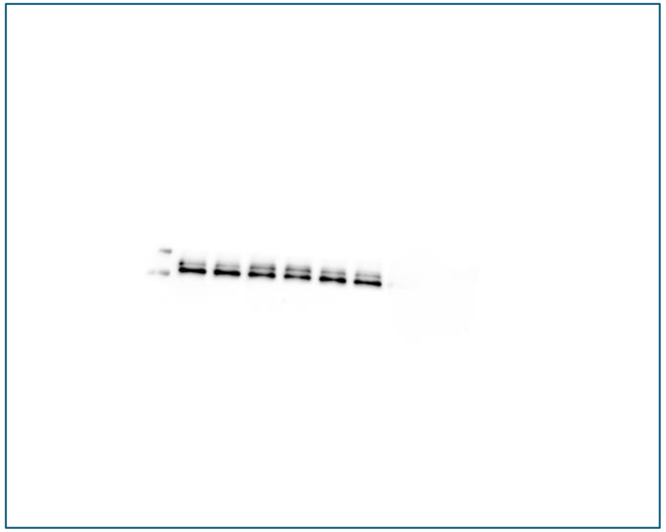

Beta actin 72 hours

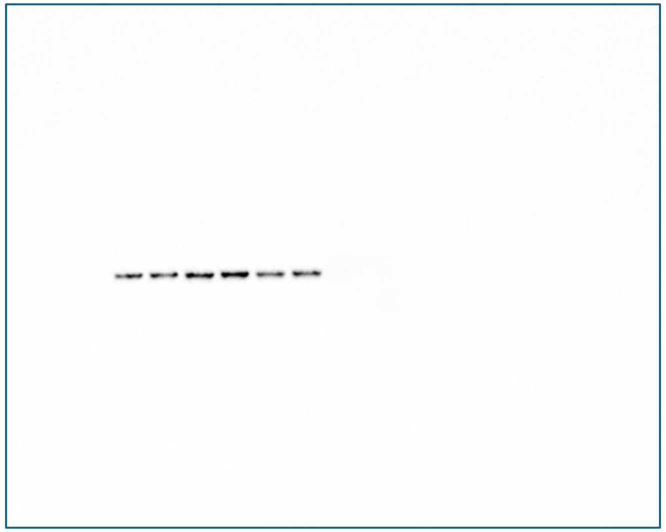

Cofilin 24 hours

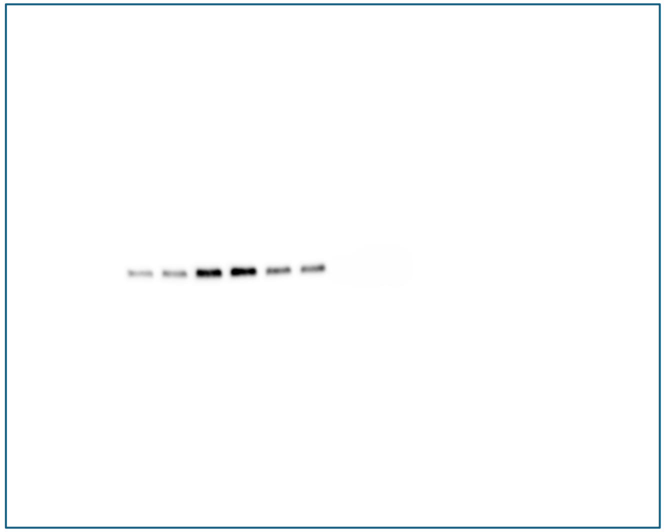

Cofilin 48 hours

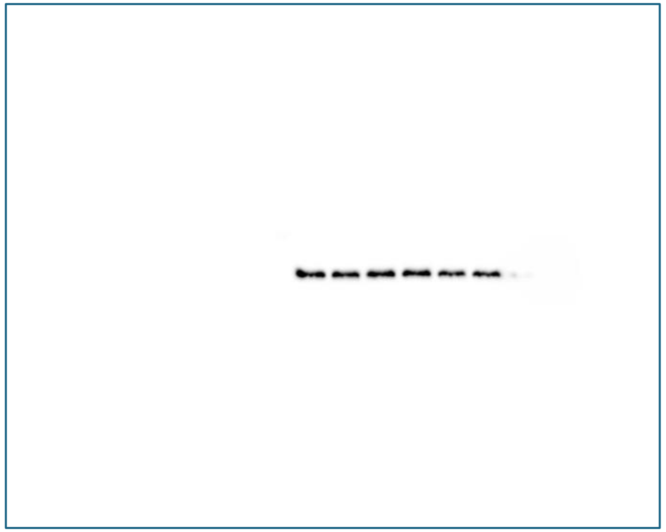

Cofilin 72 hours

Supplementary material Figure 3 B

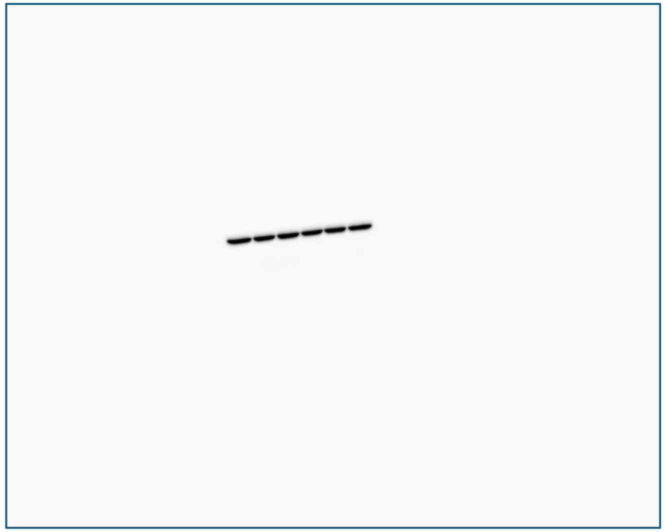

Beta actin 24 hours

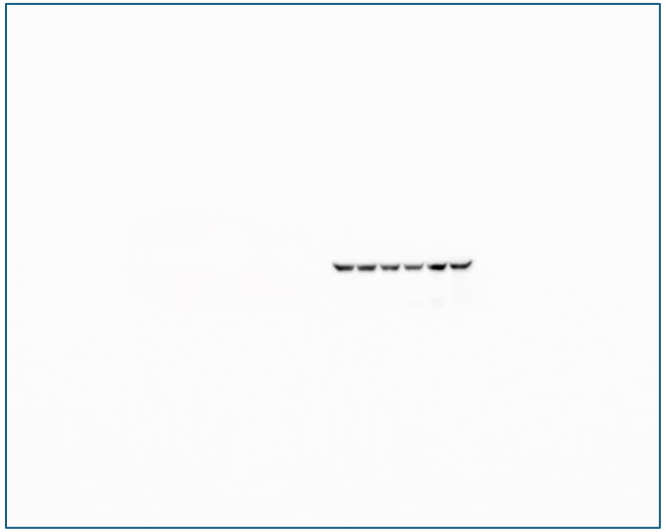

Beta actin 48 hours

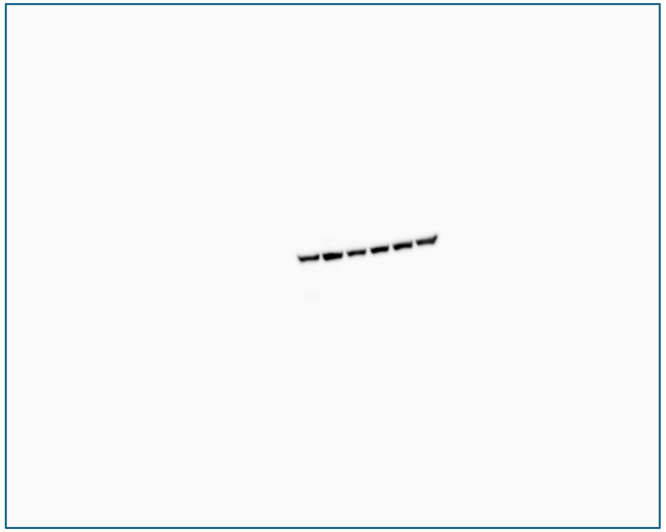

Beta actin 72 hours

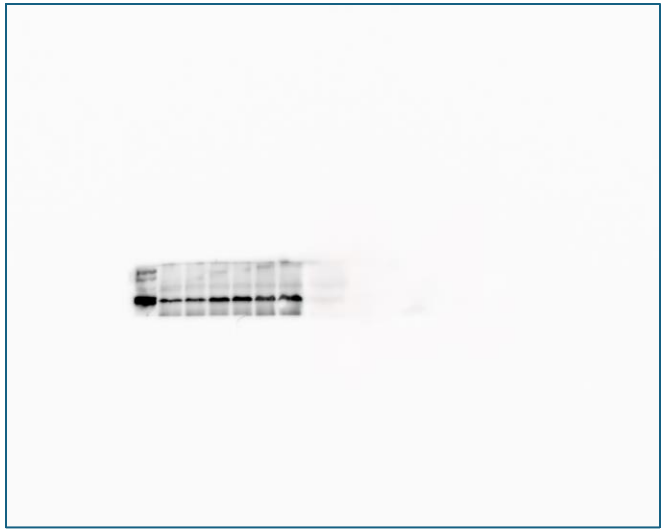

DMT-1 24 hours

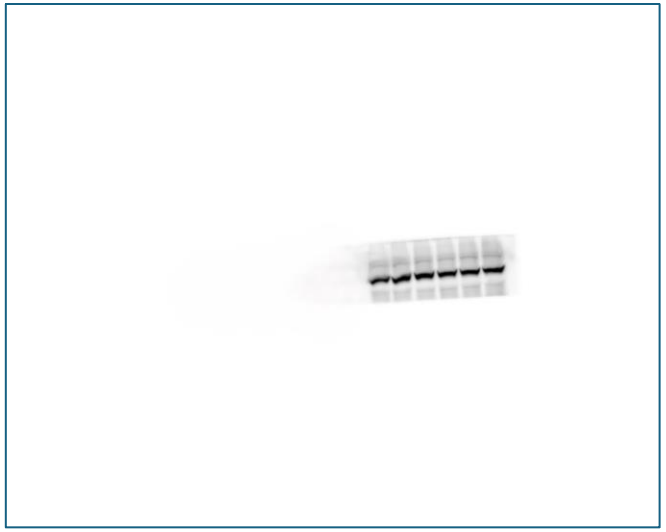

DMT-1 48 hours

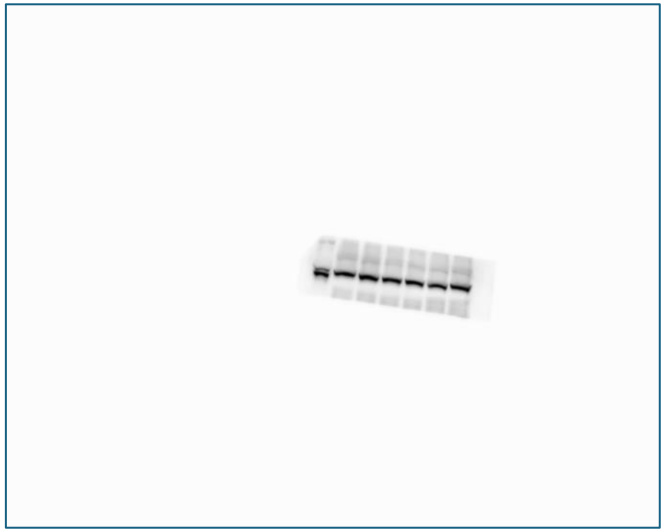

DMT-1 72 hours

Supplementary material Figure 4A

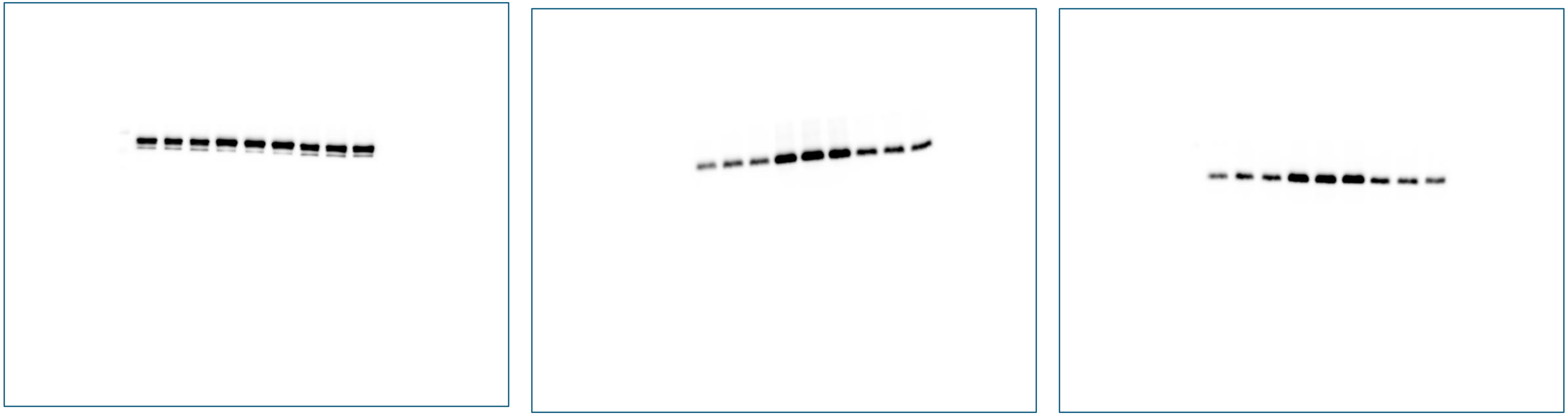

Beta Actin

pCofilin

Cofilin

Supplementary material Figure 4B

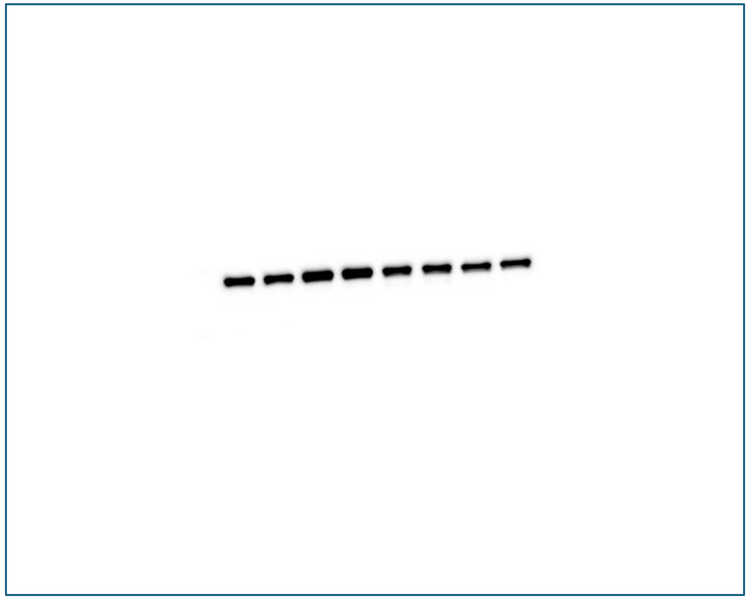

Beta Actin

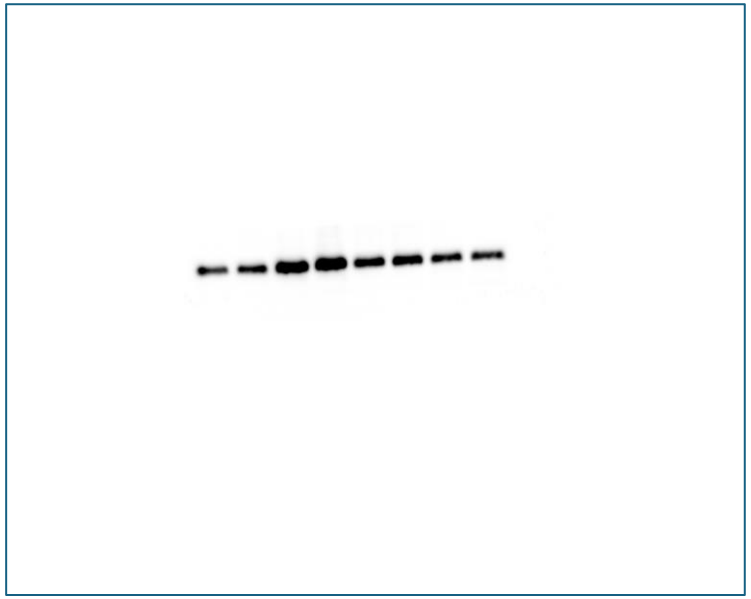

pCofilin

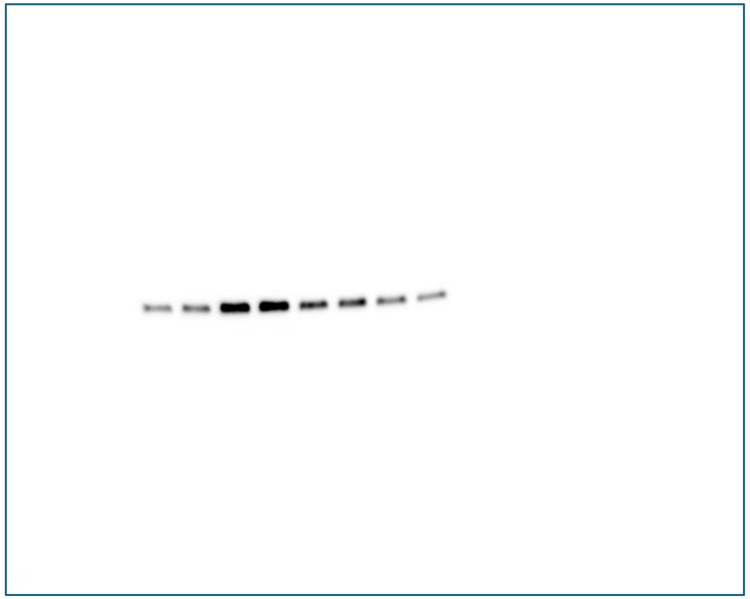

Cofilin

Supplementary material Figure 8

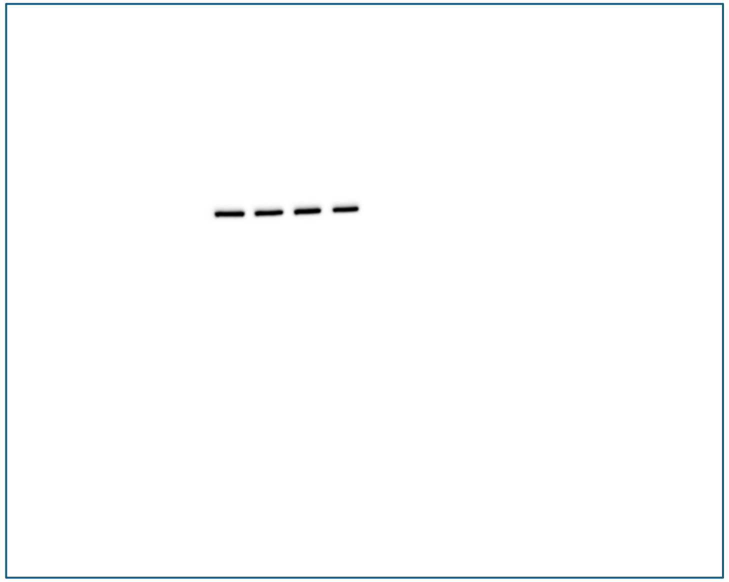

Beta Actin cytosol

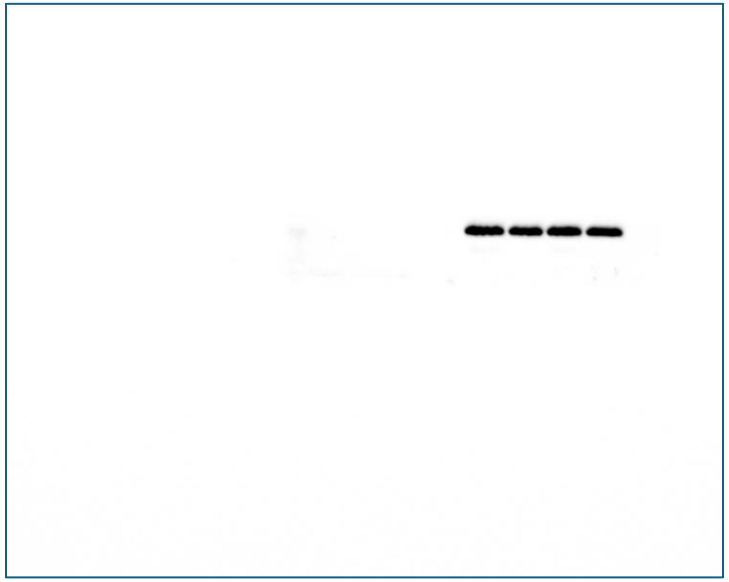

Histone Nuclear

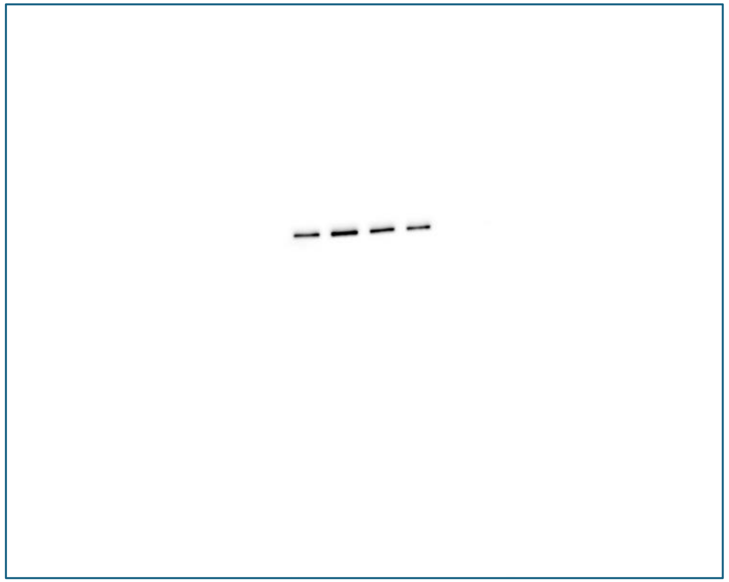

NFkB cytosol

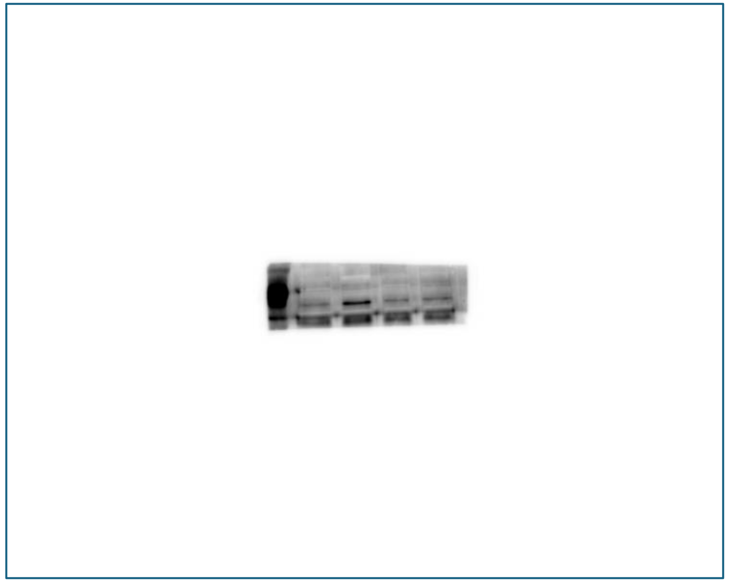

NFkB Nuclear

Certificate of analysis HMC-3 ATCC

ATCC

CERTIFICATE OF ANALYSIS

ATCC® Number: **CRL-3304™**
Lot Number: **70043235**

Name: **HMC3**
Description: **Embryonic Microglia Clone 3**
Species: **Human (*Homo sapiens*)**
Volume/Ampule: **Approximately 1 mL**
Date Frozen: **05APR2021**
Recovery: **A T-75 setup at a seeding density of 2.0 x 10^4 viable cells/cm^2 reaches approximately 40% confluence in 1 day and 90% confluence in 3 days.**
Product Format: **Cells cryopreserved in the appropriate cryopreservation medium**
Expiration Date: **Not applicable**
Storage Conditions: **Vapor phase of liquid nitrogen**

Test / Method	Specification	Result
**Ampule passage number**	Report results	Unknown + 7
**Population doubling level (PDL)**	Report results	Not applicable
**Total cells/ampule**<br>(Cell count using Trypan Blue stain method)	Report results	1.9 x 10^6 total cells/ampule
**Post-freeze viability**<br>(Cell count using Trypan Blue stain method)	≥ 50.0%	92.1%
**Growth properties**<br>(Visual observation method)	Adherent	Adherent
**Morphology**<br>(Visual observation method)	Epithelial-Like*	Epithelial-Like
**Test for mycoplasma contamination**<br>Hoechst DNA stain (indirect) method<br>Agar culture (direct) method<br>PCR-based assay	 None detected<br>None detected<br>None detected	 None detected<br>None detected<br>None detected
**Species determination: COI assay (interspecies)**	Human	Human
